# Transcriptome and proteomic analyses reveal multiple differences associated with chloroplast development in the spaceflight-induced wheat albino mutant *mta*

**DOI:** 10.1371/journal.pone.0177992

**Published:** 2017-05-24

**Authors:** Kui Shi, Jiayu Gu, Huijun Guo, Linshu Zhao, Yongdun Xie, Hongchun Xiong, Junhui Li, Shirong Zhao, Xiyun Song, Luxiang Liu

**Affiliations:** 1 School of Life Sciences, Qingdao Agricultural University, Qingdao, Shandong, China; 2 National Key Facility for Crop Gene Resources and Genetic Improvement, Institute of Crop Sciences, Chinese Academy of Agricultural Sciences, Beijing, China; University of Hyderabad School of Life Sciences, INDIA

## Abstract

Chloroplast development is an integral part of plant survival and growth, and occurs in parallel with chlorophyll biosynthesis. However, little is known about the mechanisms underlying chloroplast development in hexaploid wheat. Here, we obtained a spaceflight-induced wheat albino mutant *mta*. Chloroplast ultra-structural observation showed that chloroplasts of *mta* exhibit abnormal morphology and distribution compared to wild type. Photosynthetic pigments content was also significantly decreased in *mta*. Transcriptome and chloroplast proteome profiling of *mta* and wild type were done to identify differentially expressed genes (DEGs) and proteins (DEPs), respectively. In total 4,588 DEGs including 1,980 up- and 2,608 down-regulated, and 48 chloroplast DEPs including 15 up- and 33 down-regulated were identified in *mta*. Classification of DEGs revealed that most were involved in chloroplast development, chlorophyll biosynthesis, or photosynthesis. Besides, transcription factors such as PIF3, GLK and MYB which might participate in those pathways were also identified. The correlation analysis between DEGs and DEPs revealed that the transcript-to-protein in abundance was functioned into photosynthesis and chloroplast relevant groups. Real time qPCR analysis validated that the expression level of genes encoding photosynthetic proteins was significantly decreased in *mta*. Together, our results suggest that the molecular mechanism for albino leaf color formation in *mta* is a thoroughly regulated and complicated process. The combined analysis of transcriptome and proteome afford comprehensive information for further research on chloroplast development mechanism in wheat. And spaceflight provides a potential means for mutagenesis in crop breeding.

## Introduction

Chloroplasts originated from an endosymbiotic event between a photosynthetic cyanobacterium and a eukaryotic host [1, 2]. They are not only essential for photosynthesis, but are also responsible for the production of many important metabolites, such as amino acids, lipids, starch, hormones, vitamins and isoprenoids in higher plants [3–5]. These functions make the chloroplast an indispensable organelle for plants survival and growth. Functional and photosynthetically active chloroplasts develop from undeveloped proplastids. During this differentiation proplastids enlarge and then thylakoids are formed and stacked into defined grana [6]. Chloroplast differentiation requires the participation of many proteins. Most of these proteins are nuclear-encoded and imported into the developing chloroplast. Therefore, the nucleus regulates essential aspects of chloroplast development [7]. In addition, retrograde signaling from the developing chloroplast to the nucleus also ensures the production of appropriate levels of protein complexes involved in chloroplast maturation [3, 8].

Chlorophyll (Chl) biosynthesis occurs in parallel with chloroplast development during leaf color formation. Chl is distributed in chloroplast thylakoid membrane and, as the main part of light harvesting complex, plays an important role in photosynthesis [9]. The Chl biosynthetic pathway and Chl metabolism have been extensively studied. The enzymes and their encoded nuclear genes for all 15 biosynthetic steps have been identified [10, 11]. The leaf color and photosynthetic efficiency are directly affected by chloroplast development, the number and size of chloroplasts, and Chl biosynthesis and content [12, 13]. In addition, leaf color is also affected by temperature; relatively low temperatures lead to temporary leaf color variation in some chlorophyll-deficient mutants [14]. The ratio of chlorophylls and carotenoids alters in low temperature is most likely the reason for this. Chl includes two different forms Chl *a* and Chl *b*, which occur in an approximate ratio of 3:1, influence the leaf green color. Carotenoids are the pigments responsible for the leaf orange color [15].

Leaf color is directly related to the production of photosynthetic pigments and chloroplast development. Thus, leaf color mutants are widely used to reveal the mechanisms for the regulation of chloroplast development and function, Chl biosynthesis, and photosynthesis [12, 16–18]. Thus far, many genes and transcription factors (TFs) affecting chloroplast development and division have been identified. The phytochrome-interacting factor 3 (PIF3) functions in mediating the initial phases of light-induced chloroplast development after the first exposure of green-faded seedlings to light, through the regulation of a subset of rapidly photoresponsive nuclear genes encoding plastid and photosynthesis-related components [19]. In addition, PIF3 is a repressor negatively regulating chloroplast development in *Arabidopsis* [18]. The *Golden2-like* (*GLK*) gene family includes a pair of partially redundant nuclear TFs, GLK1 and GLK2, that are required for the expression of nuclear-encoded photosynthetic genes and chloroplast development in diverse plant species, including maize (*Zea mays*), rice (*Oryza sativa*), and *Arabidopsis thaliana* [2, 20–22]. As in rice, *GLK1* and *GLK2* are expressed in partially overlapping domains in photosynthetic tissue, the *glk1glk2* double mutant is pale green in all photosynthetic tissues and there is a reduction in grana thylakoids in the chloroplasts [20]. FtsZ is a key structural component of chloroplast division machinery in perhaps all photosynthetic eukaryotes. The chloroplast forms of FtsZ assemble into inner membrane-associated rings in the stromal compartments [23]. Members of the *Accumulation and Replication of Chloroplast* (*ARC*) gene family cooperate with FtsZ to regulate chloroplast division [24]. Besides, The three isoforms of NADPH: protochlorophyllide oxidoreductase (POR), PORA, PORB and PORC, are the key enzyme catalyzing Chl biosynthesis. *PORA* is expressed primarily early in development including etiolation, germination and greening. *PORB* is expressed throughout leaf. *PORC* expression pattern is similar to *PORB* when treated by high light [17, 25].

Despite knowledge of the genes involved in model plant species, the molecular mechanisms for chloroplast development and Chl biosynthesis are not well-understood in hexaploid wheat due to its large genome. In species such as wheat large-scale transcriptome profiling and chloroplast proteome analysis (2D-DIGE and MALDI-TOF /TOF) will be helpful in obtaining a global view of gene expression patterns and provide insight into the potential molecular mechanisms of mutagenesis generating [13, 26, 27]. Transcriptome database are valuable resources for genetic and genomic studies of multiple difference genes and pathways. For example, genes involved in chloroplast development and division, Chl biosynthesis, and pigment biosynthesis and transport can be identified via transcriptome analysis [13]. And wheat proteomic studies with a special focus on chloroplasts provide a better understanding of the proteins involved in photosynthesis [27].

We have obtained a novel chlorophyll-deficient mutant *mta* from the progeny of Mt6172 (MT), generating from the wild type (WT) *Triticum aestivum* L. H6172, which was exposed to spaceflight induction [14]. To explore the genetic alterations under space environment and uncover the mechanisms underlie the albino phenotype of *mta*, we performed chloroplast ultra-structural observation and photosynthetic pigments assays of *mta* and WT. We found that chloroplasts in *mta* exhibited distorted morphology and were less widely distributed than that of WT. The photosynthetic pigment content was significantly decreased in *mta*. Leaf transcriptome sequencing and chloroplast proteomic analyses of *mta* and WT were also carried out. The DEGs and DEPs associated with chloroplast components and development, Chl biosynthesis, and photosynthesis were identified. Omics data mining revealed new metabolic pathways and TFs involved in leaf color formation. Importantly, we found that the DEPs were significantly functioned in photosynthesis including PSⅠ, PSⅡ, cytochrome b6f complex and F-type ATPase. The expression levels of selected DEGs and genes encoding DEPs were validated by qRT-PCR. Integrated omics analyses revealed the main molecular mechanisms regulating leaf color formation, involving chloroplast development, chlorophyll biosynthesis, and photosynthesis. These findings provide new clues to binding omics means for discovering multiple differences in mutagenesis on polyploidy plant species. In addition, spaceflight provides an approach to generating mutants that can be used in crop breeding research.

## Materials and methods

### Plant materials

All wheat seeds were kindly selected and provided by Space Breeding Research Center, Chinese Academy of Agricultural Sciences. The WT and MT wheat seeds were grown in an experimental field at the Chinese Academy of Agricultural Sciences, Beijing for three weeks. At their three leaf stage, the whole leaves of WT and *mta* plants were separately collected for transcriptome sequencing analysis. The same plants leaves of WT and *mta* were used for chloroplast protein extraction and identification. For qRT-PCR validation, the leaves powder for RNA-seq were collected in tubes, frozen in liquid nitrogen, and then stored at -80°C until analysis.

### Transmission electron microscopy (TEM)

The three-week-old seedling leaves of WT and *mta* were cut into 1×1 mm pieces and fixed in 2.5% glutaraldehyde for 24 hours at 4°C. After washing with 0.1 mol/L phosphate buffer (pH 7.2), leaf samples were fixed in 1% osmium tetroxide (OSO_4_) for 2 hours and washed with phosphate buffer again. Tissues were dehydrated through an ethanol series, 30, 50, 70, 80, 90, 95, and 100%, successively. Then embedded in Epon 812 and sectioned using a Leica ultramicrotome (Leica Microsystems, Ltd, Germany). After staining in 0.2% lead citrate, the ultrastructure of leaf cells was observed under a transmission electron microscope (HT-7700, Hitachi, Japan).

### Contents of chlorophyll and carotenoid assays

A total of 0.2 g fresh leaves from WT and *mta* were cut into 2×2 mm pieces and submerged in 10 ml 95% alcohol for 24 hours in the dark with five replicates, respectively. Leaf extractions were made after swirled and oscillated the samples for 6–8 times until leaves turned white. The specific light absorption of leaf extracts containing chloroplast pigments were measured at 665, 649, and 470 nm using a SPECORD 200 spectrophotometer (AnalytikJena, Germany) according to a previously published method [28, 29]. Chlorophyll and carotenoid content was calculated as following formulas: Chl a = 13.95 × A_665_ − 6.88 × A_649_, Chl b = 24.96 × A_649_ − 7.32 × A_665_, Car = (1000A_470_ − 2.05Chl a − 114.8Chl b)/245.

### RNA isolation and library preparation

All leaves of 80 WT and 70 *mta* plants were collected and a total of four libraries with two biological replicates were prepared. RNA was extracted using TRIzol-A^+^ reagent (TIANGEN BIOTECH, Beijing) and treated with RNase-free DNase I (TaKaRa). RNA quantity was measured using a Nanodrop Quibt 2.0 Flurometer (Life Technologies, CA, USA). RNA quality was evaluated with an Agilent Bioanalyzer Model 2100 (Agilent Technologies, Palo Alto, CA). Samples with an RNA Integrity Number (RIN) value greater than 6.4 were deemed acceptable according to the Illumina transcriptome sequencing protocol. All sequencing reads from the four libraries have been submitted to the Sequence Read Archive (SRA), National Center for Biotechnology Information (NCBI) with an accession of SRP106763 (BioProject ID of PRJNA386075; Four run ID of SRR5520556, SRR5527113, SRR5527114 and SRR5527116 represent *mta*_R1, *mta*_R2, WT_R1 and WT_R2 sample reads, respectively).

### Transcriptome sequencing and assembly

WT and *mta* poly (A) mRNA were enriched using oligo dT magnetic beads, then fragmented using fragmentation buffer. The fragmented mRNA was used as template for cDNA synthesis. First strand cDNA was synthesized using reverse transcriptase and random primers, followed by second strand cDNA synthesis using DNA PolymeraseI and RNaseH. Samples were cleaned using the QIAquick PCR kit, and were eluted by eluent buffer for end repairing and sequencing adapter joining. Then the cDNA fragments were separated by agarose gel electrophoresis and fragments of 100–300 bp were enriched by PCR amplification to create cDNA libraries. The well-constructed cDNA libraries were then sequenced on the Illumina HiSeq 2500 platform. The fastq format raw reads were first processed using in-house perl scripts. In this step, clean reads were obtained by removing reads containing adapter, ploy-N, and low quality reads from raw reads. At the same time, Q30 and GC-content of the clean data were calculated. Assembly of the clean reads was performed using Trinity [30], according to the reference genome Chinese Spring wheat in the public databases.

### Functional annotation and DEG identification

Gene function was annotated based on the following databases: NCBI non-redundant protein sequences (NR), Protein family (Pfam), UniProt/Swiss-Prot, Gene Ontology (GO), Clusters of Orthologous Groups of proteins (KOG/COG), and Kyoto Encyclopedia of Genes and Genomes (KEGG). Unigene expression levels were expressed as fragments per kilobase of transcript per million fragments mapped (FPKM). FPKM values were calculated using RSEM [31]. Differential expression analysis and identification of DEGs between WT and *mta* were performed using the DEGseq R package, which provides a statistical method for determining differential expression using a model based on the negative binomial distribution [32]. The resulting p-values were adjusted using the Benjamini and Hochberg’s approach for controlling the false discovery rate (FDR). Unigenes with an adjusted p-value ≤ 0.01 and **|**log_2_ fold changes| ≥ 1 between *mta* and WT were designated DEGs. These DEGs were also annotated with GO, COG and KEGG assignments to obtain significantly enriched pathways based on DEG-enrichment by the right sided fisher exact test [33].

### Chloroplast protein extraction

Chloroplasts were isolated from WT and *mta* following a modified protocol [34]. Each step was performed at 4°C or on ice. Briefly, 20 g fresh seedling leaves for each sample were harvested and homogenized with a mortar and pestle in extraction buffer. The homogenate was filtered through 6 layers of muslin, and this step was repeated twice. After that, the suspension containing chloroplasts was subsided by centrifugation, and the pellets containing the chloroplasts were resuspended in isolation buffer. The above resuspended material was then loaded on top of a Percoll step gradient. After centrifugation, the chloroplasts were isolated and from washed twice with isolation buffer and then directly used for protein extraction after visualization under a fluorescence microscope (OLYMPUS, U-RFL-T, Germany) to assess quality. Total chloroplast protein was extracted using TCA/acetone precipitation [35], and chloroplast pellets were resuspended in suspension buffer [36]. Protein concentration was measured with the Bio-Rad Protein Assay kit, which was based on the Bradford method using Bovine Serum Albumin (BSA) as a standard. Proteins were purified using a 2D Clean-up kit (GE Healthcare, USA) with all steps performed on ice, and all chloroplast proteins were dissolved in DIGE lysis-buffer (labeling buffer). Next, the Bradford method was again used to measure the protein concentration for quantitative analysis of the whole leave chloroplast proteins.

### 2D-DIGE and image analyses

Two-Dimensional Difference Gel Electrophoresis (2D-DIGE) was carried out after analytical gels labeling WT and *mta* chloroplast proteins with Cy2 (blue), Cy3 (green) and Cy5 (red) fluorescent dyes (5 nmol Cyanine Dye DIGE Fluor mimimal Dye labeling kit, GE, USA). Preparative gels staining with coomassie blue G-250. All steps were done according to the manufacturer’s instructions. The experimental strategy is shown in **S1 Fig**. Three biological replicate WT samples (50 μg each) were labeled with Cy3 (one sample) and Cy5 (two samples), and the *mta* samples (50 μg each) were labeled with Cy3 (two samples) and Cy5 (one sample). The labeled samples were combined and separated on 2-DE gels together with the internal standard (IS), which was prepared by mixing 25 μg WT and 25 μg *mta* samples and labeling with Cy2. Labeling reactions were carried out according to the manufacturer’s instructions. Isoelectric focusing (IEF) was performed using Ettan IPGphor according to GE Healthcare operating manual and a previously described method [37]. All gels were scanned using a scanner (GE Healthcare, USA) according to the manufacturer's protocol. The abundance of each protein spot in the scanned images was quantified using Image Master Platinum 7.0 software (GE Healthcare, USA).

### Protein identification by MALDI-TOF MS

All selected spots were manually excised from the WT and *mta* chloroplast proteins 2D-DIGE gels. The normalized volume of each spot was assumed to represent the abundance of the detected protein. A criterion of ≥ 1.5-fold change (1.5-fold increase / decrease, p-value ≤ 0.05) was used to define significant differences when comparing spot sizes between groups. In-gel digestion and MS acquisition were performed as described [38]. TOF mass spectra (TOF-MS) were searched against the NCBInr (http://www.ncbi.nlm.nih.gov/) protein database using the MASCOT search engine (http://www.matrixscience.com, Matrix Science). Identification was based on the combination of a MASCOT score, maximum peptide coverage, and additional experimental pI and MW of the protein spots in the gels. The NCBI and TAIR (http://www.arabidopsis.org/) database were used to obtain information about protein functional annotation, encoding genes and subcellular localization.

### Bisulfite sequencing PCR (BSP)

BSP was utilized to determine the methylation status at single CpG resolution of *TaPORA* promoter fragments. Genomic DNA was isolated from the leaves of WT and *mta* using a DNA-quick Plant System kit (Tiangen Biotech, Beijing, China) according to the manufacturer’s instructions. Approximately 500 ng of genomic DNA were treated with bisulfate CT conversion reagent following the protocols in the EZ DNA Methylation-Gold ^TM^ kit (ZYMO research corp, USA). *TaPORA* promoter primers for BSP (**S5 Table**) were designed using the online tool MethPrimer (http://www.urogene.org/methprimer/). BSP reaction with 25 μl per tube was done using high fidelity PrimeSTAR GXL DNA polymerase (Takara Biotech, Dalian, China) and BSP primers. The -PCR protocols were as follows: 98°C for 2 min; 35 cycles of 98°C for 10 s, 57°C for 15 s, and 68°C for 20 s; and a final extension 68°C for 6 min. The PCR products were separated by electrophoresis on 2% agarose gels and were then purified using a gel extraction kit (Axygen Biotech, Hangzhou, China) following standard protocols. The purified fragments were cloned into the pLB vector (Tiangen Biotech) and verified by sequencing in Sangon Company (Shanghai, China). At least five clones were sequenced for each BSP reaction.

### Quantitative real-time PCR (qRT-PCR) validation

qRT-PCR analysis using the Bio-Rad CFX Manager (Bio-Rad, USA) with SsoFastTM EvaGreen Supermix (Bio-Rad) was employed to verify the DEG and DEP expression results. Primers for specific genes encoding photosynthetic proteins and photosynthesis DEGs (**S6 Table**) were designed using Beacon Designer 7 (Bio-Rad, USA). qRT-PCR assays were performed in triplicate (technical repeats) with two independent biological replicates, and *ACTIN* (GenBank: AAW78915) was used as theIS [39]. Relative expression levels were determined using a relative quantitative method (2^−ΔΔCT^) [40].

### Statistical analysis

Statistical analysis was performed by SAS8.01 software (SAS Institute, Cary, NC, USA). The data of Chl and carotenoid contents were expressed as the mean ± standard deviation. All data were analyzed using one-way ANOVA. The p-value 0.05 and 0.01 represent different significant level with confidence of 95% and 99%, respectively.

## Results

### Phenotypic properties of mta: Leaf color and chloroplasts

**Fig 1:**
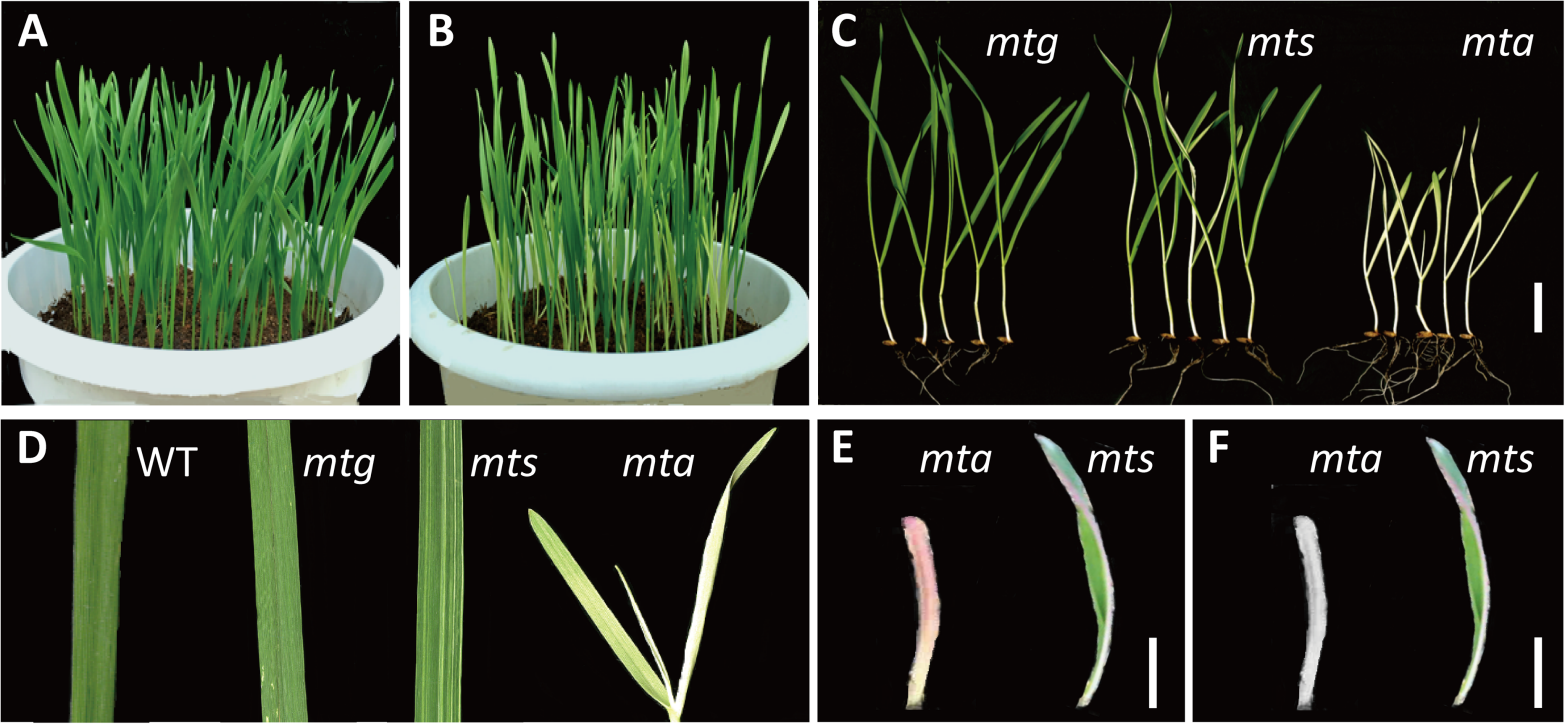
Phenotype of wild type and leaf color mutants of *Triticum aestivum* L. (A) Wild type; (B) Leaf color mutants; (C) Type of leaf color mutants: *mtg*, green leaf; *mts*, narrow-white striped leaf; *mta*, albino leaf. (D) Leaf of wild type and mutants. (E) Phenotype of *mta* and *mts* white tissue in low temperature; (F) Phenotype of *mta* and *mts* white tissue as temperature increased. (All white bar = 2 cm).

Mt6172 (MT) was a spaceflight-induced mutant originating from a winter wheat cultivar, H6172 (WT) (**Fig 1A**). The self-fertilized progenies of MT segregated three types of leaf color: green, narrow-white striped and albino. These mutants were named *mtg*, *mts* and *mta*, respectively (**Fig 1B and 1C**). The *mta* mutant had completely albino leaves and died after 25–28 days. The white leaf tissue in *mts* and *mta* turned pink or purple when temperature decreased to 4–10°C, but returned to albino as temperature increased (**Fig 1D–1F**). Although transiently affected by an environmental factor, *mta* leaf color of *mta* was mainly controlled by genetic regulation.

**Fig 2:**
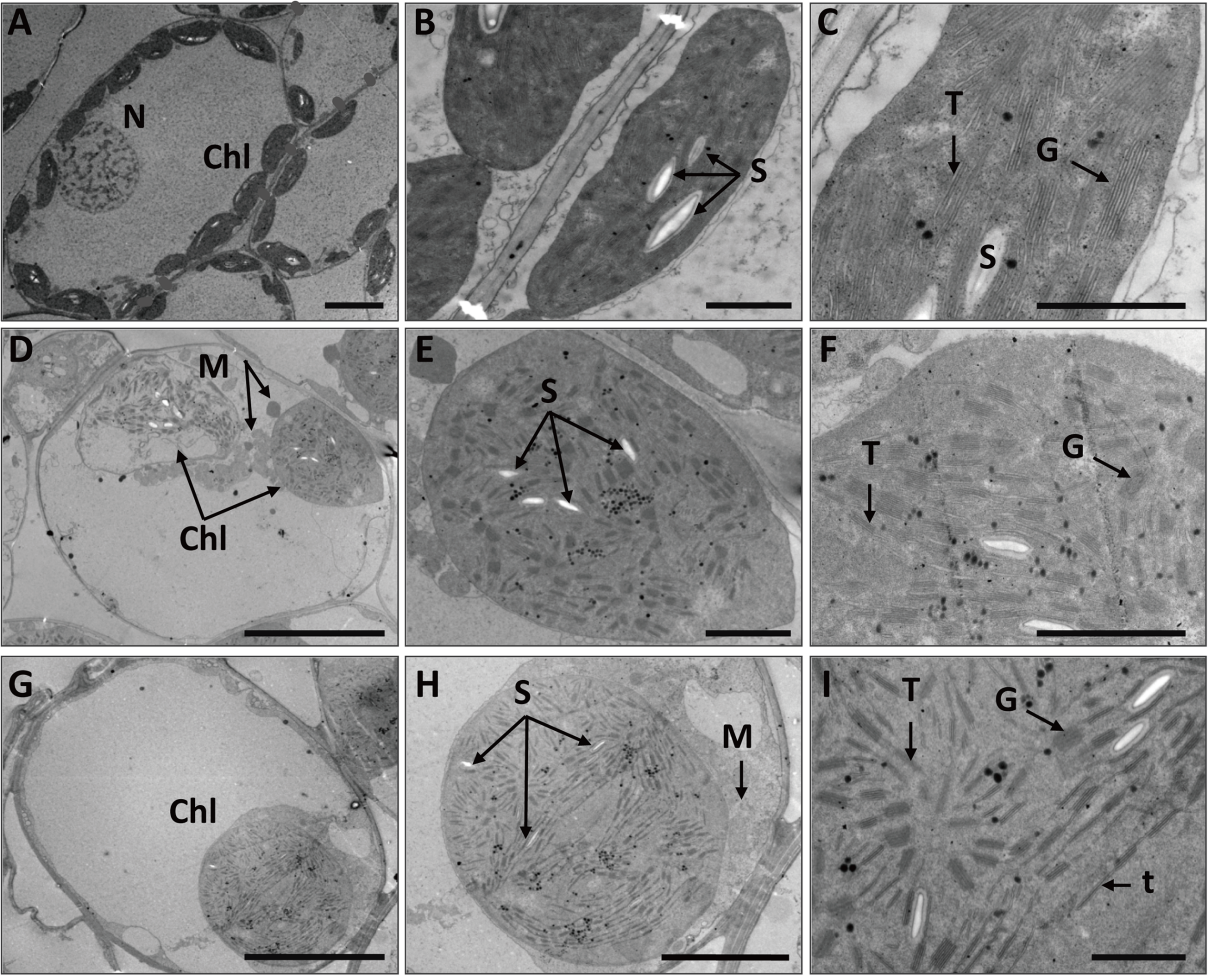
Chloroplast ultrastructures of WT and *mta*. (A-C) Chloroplast ultrastructure of WT (A, bar = 10 μm; B, bar = 2 μm; C, bar = 1 μm); (D-I) Chloroplast ultrastructure of *mta* (D, G bar = 10 μm; E, bar = 2 μm; F, bar = 1 μm; H, bar = 5 μm; I, bar = 1 μm). In these pictures, Chl, denotes chloroplast; N, denotes nucleus; M, denotes mitochondria; S, denotes starch; T, denotes thylakoid grana; G, denotes grana; t, denotes single thylakoid sac.

To investigate chloroplast morphology and distribution in *mta*, we further analyzed the chloroplast ultrastructure of *mta* and WT. As shown in **Fig 2**, WT chloroplasts exhibited typical structures and had a highly organized inner membrane system, numerous granum and inter-granum thylakoids and several starch granules (**Fig 2A–2C**). In *mta*, mesophyll cells contained rarely or none chloroplasts, and the chloroplasts were abnormal appearing inflated (**Fig 2D–2I**). The inner structure of inflated chloroplasts almost disappeared and contained disordered granum lamellae. These chloroplasts had smaller and more abundant starch granules than those in WT (**Fig 2D–2F**). Another large-sized chloroplasts in *mta* had no intact membrane structures, and contained many single thylakoid sacs and small starch granules (**Fig 2G–2I**). These results confirm that chloroplast development in *mta* is impaired.

Chl and carotenoid content were also significantly different between *mta* and WT (**Table 1**). Total Chl, Chl *a*, Chl *b*, and carotenoid content in *mta* were much lower than in WT. The ratio of Chl *a*/Chl *b* in *mta* was very close to WT, but the ratio of carotenoid/Chl in *mta* was slightly higher than that of WT, indicating that the total Chl content decreased more than the carotenoid content in *mta*. These data suggest that albino leaf phenotype of *mta* is directly affected by decreased pigment content.

**Table 1:** Chlorophyll and carotenoid content in WT and *mta*.

Sample	Chl a (mg·g^-1^)	Chl b (mg·g^-1^)	Chl a + b (mg·g^-1^)	Chl a/b	Carotenoid (mg·g^-1^)	Carotenoid/Chl
WT	1.19 ± 0.06	0.29 ± 0.02	1.48 ± 0.07	4.06 ± 0.15	0.30 ± 0.02	0.20 ± 0.01
*mta*	0.19 ± 0.17^a^	0.05 ± 0.01^b^	0.24 ± 0.03^a^	4.00 ± 0.26^a^	0.06 ± 0.01^a^	0.24 ± 0.01^a^

^a^ Significant difference (P≤0.05)

^b^ Significant difference (P≤0.01).

### Transcriptome analysis

#### Illumina sequencing and unigenes assembly

To gain insight into the mechanism of abnormal chloroplast development in *mta*, we used transcriptome analysis to identify chloroplast-related genes that are differentially expressed between WT and *mta*. Four cDNA libraries yielded 30,608,728 to 78,882,496 pair-end clean reads (S1 Table). The total reads were mapped to the reference Chinese Spring genome (*Triticum aestivum* IWGSC1_popseq.31). Using Trinity, the clean reads of WT and *mta* were assembled into 105,265 transcripts (Table 2), which comprised 64,999 unigenes. The length of 44.85% of the transcripts ranged from 300 bp to 1000 bp, 21.2% were shorter than 300 bp and 33.92% were longer than 1000 bp. These results indicated that clean data was in good quality. We therefore used these data for the following analyses.

**Table 2:** Summary of functional annotations and length distributions of unigenes and DEGs between WT and *mta*.

	Total	GO	COG	KEGG	NR	Swiss-Prot
**All unigenes**						
Unigenes	105,256	89,470	36,855	37,883	105,214	77,679
Length < 300	22,339	18,420	4,356	7,346	22,339	13,698
300 ≤ Length < 1000	47,211	39,777	16,797	17,393	47,185	34,732
1000 ≤ Length	35,706	31,273	15,702	13,144	35,700	29,249
**DEG Set**						
*mta* / WT	4,588	4,065	2,583	1,703	4,498	3,844
*Mta*_R1 / WT_R1	6,191	5,468	2,443	2,251	6,060	5,143
*Mta*_R2 / WT_R2	6,421	5,673	2,497	2,501	6,417	5,143

#### Unigenes functional annotation and DEG classification

To obtain transcript functional annotations, all assembled genes were searched against five public databases (GO, COG, KEGG, Swiss-Prot, and NR). The correlation coefficients for both the WT and *mta* biological replicates reached significant level and were higher than 0.96 (S2 Fig). This result demonstrated that the unigenes were suitable for further analysis. Among these unigenes, a total of 4,588 DEGs were identified between WT and *mta*. The summary of DEGs functional annotation and classification were provided in Table 2.

**Fig 3:**
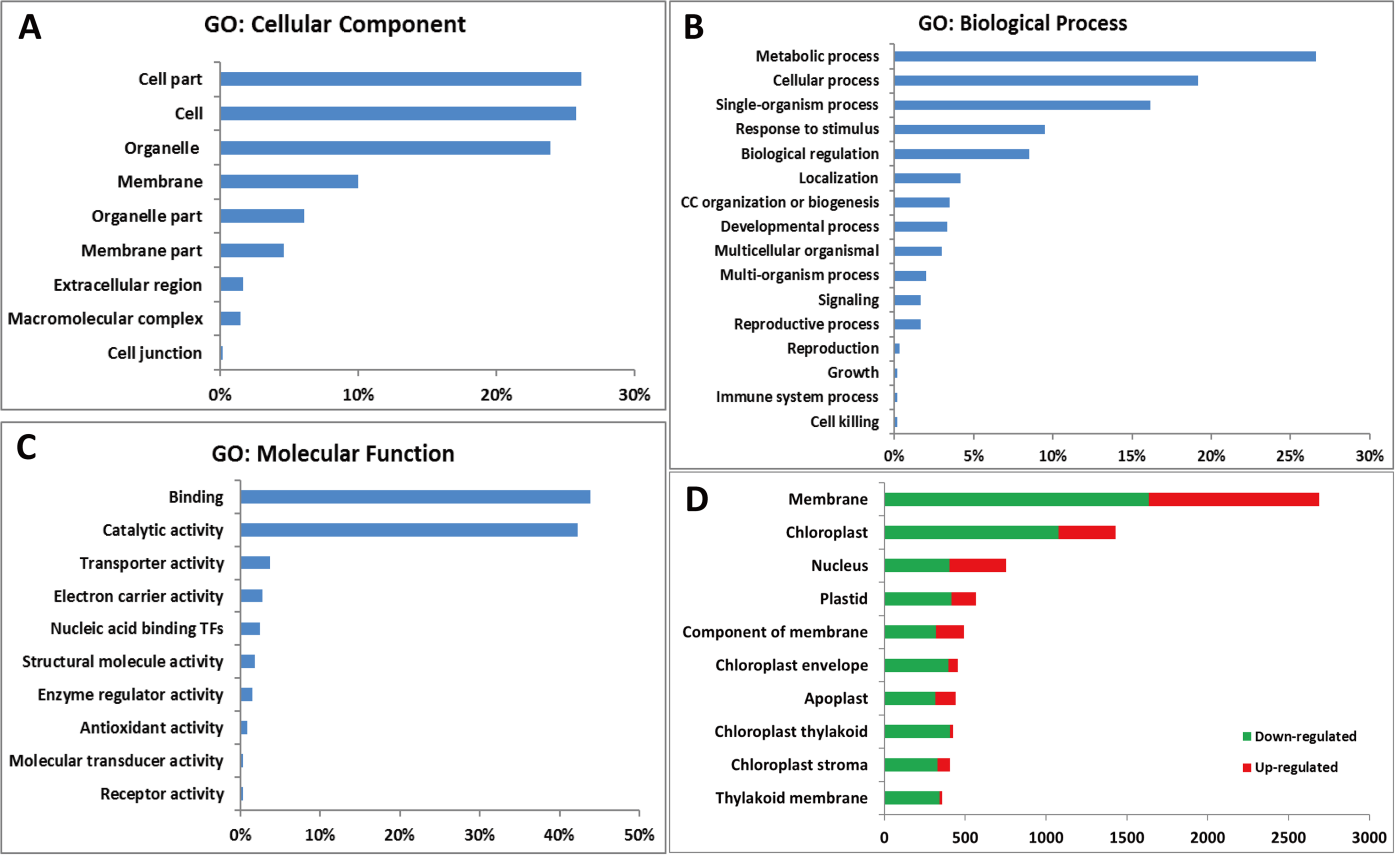
Functional categorization of DEGs between WT and *mta* based on GO annotation. The proportion of each category is displayed based on (A) cellular component, (B) biological process, or (C) molecular function. (D) The number of DEGs in different cellular component categories, green color represents down-regulated in *mta* while red color represents up-regulated in *mta*.

GO annotation of all 4,588 DEGs was performed using the WEGO software [41] (http://www.geneontology.org/), and 4,065 DEGs were categorized into cellular component, biological process and molecular function using blast topGO bio-conductor (http://www.blast2go.org/) (**Fig 3**). The cellular components category mainly included DEGs related to cell part, cell, organelle part, and membrane (**Fig 3A**). Biological processes category was classified as metabolic term (27%), cellular term (19%), single-organism process (16%) and other processes (**Fig 3B**). Molecular functions contained 44% DEGs in binding function and 42% in catalytic activity (**Fig 3C**). Further analysis of different cellular components showed that DEGs were significantly enriched in membrane, chloroplast and nucleus, and most DEGs were down-regulated in *mta*. Specially, almost all the DEGs involved in chloroplast thylakoid and thylakoid membrane were down-regulated (**Fig 3D**).

**Fig 4:**
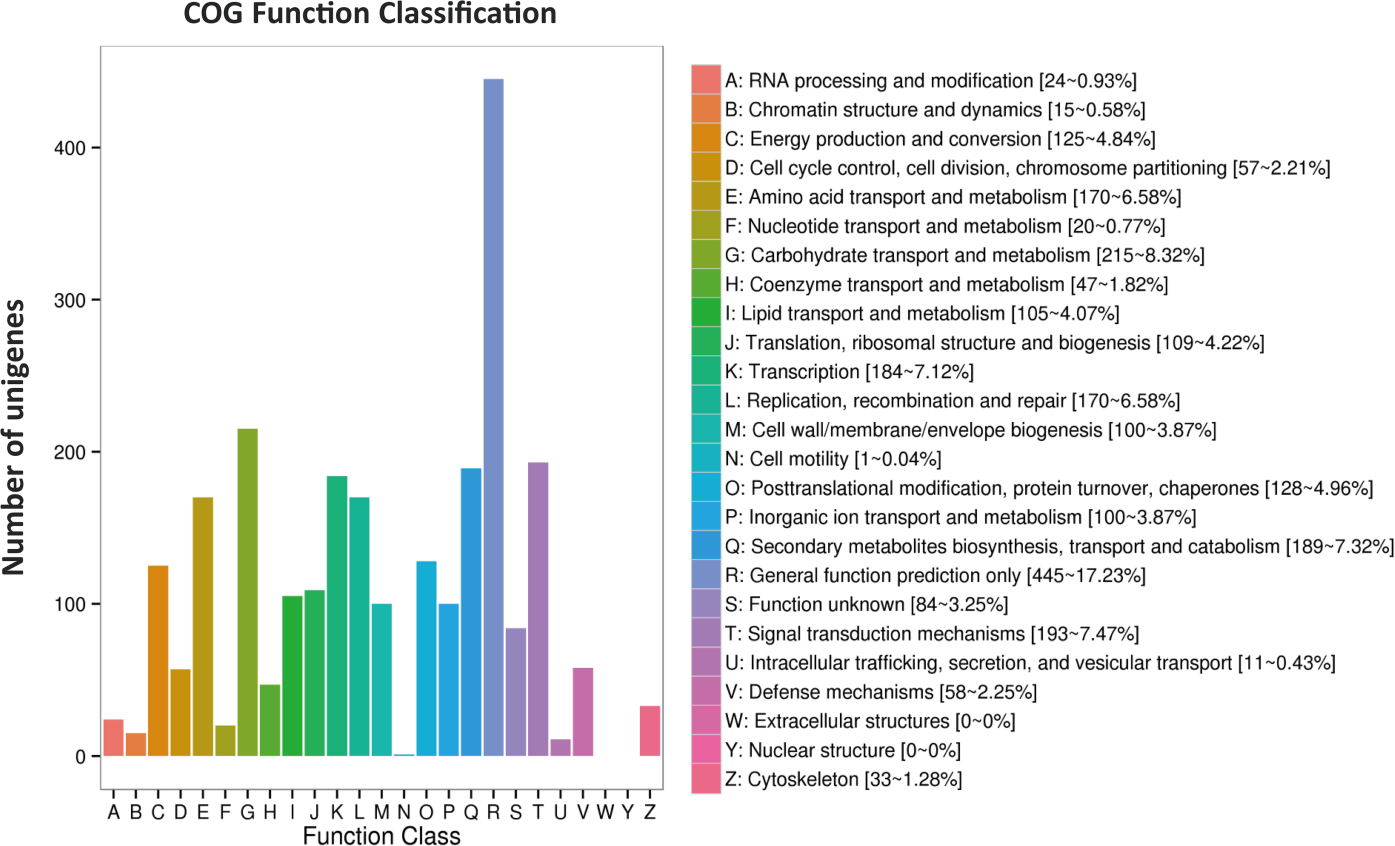
Histogram of COG classifications. 2,583 DEGs were grouped into 24 COG categories.

All 4,588 DEGs were further evaluated based on COG annotation. A total of 2,583 (56.30%) were clustered into 24 COG categories (**Fig 4**). The “general functional prediction only” cluster (445, 17.23%) represented the largest group, followed by “carbohydrate transport and metabolism” (215, 8.32%), “signal transduction mechanisms” (193, 7.47%), “amino acid transport and metabolism” (170, 6.58%), “posttranslational modification, protein turnover, chaperones” (128, 4.96%), “energy production and conversion” (162, 4.57%), “cell wall/ membrane/ envelope biogenesis” (100, 3.87%), “cytoskeleton” (33, 1.28%), and “RNA processing and modification” (24, 0.93%). No genes were assigned to “extracellular structures” and “nuclear structure”.

In order to reconstruct the metabolic pathways that might be involved in chloroplast development, Chl biosynthesis and photosynthesis, all of the DEGs were searched against KEGG database (http://www.genome.jp/kegg/) and mapped onto 111 KEGG pathways. The top 20 enriched pathways were selected (**Table 3**). Among these pathways, “phenylalanine metabolism”, “carbon fixation in photosynthetic organisms”, “porphyrin and chlorophyll metabolism”, “photosynthesis”, and “photosynthesis- antenna proteins” were significantly enriched with a q-value cut-off of 2.20×E^-49^.

**Table 3:** The top 20 significantly enriched pathways identified by KEGG analysis.

Rank	KEGG pathway term	Gene number	Rich factor ^a^	q-value ^b^	Pathway ID
1	Phenylalanine metabolism	111	2.029766	0.00E^+00^	ko00360
2	Carbon fixation in photosynthetic organisms	88	2.958113	0.00E^+00^	ko00710
3	Porphyrin and chlorophyll metabolism	62	4.840904	0.00E^+00^	ko00860
4	Photosynthesis	75	8.138023	2.20E^-49^	ko00195
	Photosynthesis—antenna proteins				ko00196
5	Glyoxylate and dicarboxylate metabolism	61	2.736091	1.30E^-10^	ko00630
6	Carbon metabolism	137	1.866761	4.04E^-10^	ko01200
7	Phenylpropanoid biosynthesis	149	1.957957	1.19E^-09^	ko00940
8	Monoterpenoid biosynthesis	20	4.838344	1.72E^-07^	ko00902
9	Cutin, suberine and wax biosynthesis	29	3.291935	1.03E^-06^	ko00073
10	Starch and sucrose metabolism	104	1.697702	7.79E^-06^	ko00500
11	Pentose phosphate pathway	35	2.402294	1.32E^-04^	ko00030
12	Glycolysis / Gluconeogenesis	69	1.749535	4.53E^-04^	ko00010
13	Fatty acid elongation	32	2.372977	4.86E^-04^	ko00062
14	Glutathione metabolism	57	1.848673	5.96E^-04^	ko00480
15	Thiamine metabolism	14	3.562022	2.68E^-03^	ko00730
16	Fatty acid metabolism	43	1.787461	1.75E^-02^	ko01212
17	alpha-Linolenic acid metabolism	34	1.900516	2.62E^-02^	ko00592
18	Limonene and pinene degradation	10	3.689237	2.83E^-02^	ko00903
19	Nitrogen metabolism	26	1.937781	1.01E^-01^	ko00910
20	Plant hormone signal transduction	76	1.430521	1.14E^-01^	ko04075

^a^ Reflect the enrichment level of DEGs on pathway, the higher number shows more significant

^b^ Multiply hypothesis testing calibration of p-value, the top 20 significantly enriched pathways were selected based on q-value.

#### Identification of DEGs related to chloroplast development and chlorophyll biosynthesis

Based on the DEGs annotation and classification above, we found genes involving in the chloroplast development and division, Chl and pigment biosynthesis, and other TFs were all included in the transcriptome database. These gene expression levels in *mta* were significantly different from those in WT (**Table 4 and S2 Table**). Many genes and TFs regulated chloroplast development were found in *mta*, three genes Triticum aestivumLinn_newGene_8077, Traes5AS_049A22D30, and Traes5AS_EBCEA6601 annotated as transcription factor PIF3 were up-regulated. The GLK family including TRAES3BF062500050CFD_g, Traes_7AL_6279FCE8B and Traes_7DL_58DDB90E3 was down-regulated. Chloroplast division gene *FtsZ* (Traes2AL_6EE01D4C2) was also down-regulated in *mta*. DEGs participating in porphyrin and chlorophyll metabolism (**S3 Fig**), such as *HEMA1*, *CHLD*, *GUN4*, *CHLI1*, *CRD1*, *PORA*, and *PORB* were all down-regulated, except *HEME1*, which was slightly up regulated in *mta*. Among all those DEGs, which were identified as involving in chlorophyll biosynthesis, the *PORA* gene expression level was much lower than other genes (**S2 Table**). Methylated CpG sites in the *TaPORA* promoter were predicted in CpGisland (http://www.urogene.org/). The methylation level of *TaPORA* promoter was further detected, and result showed that the methylation level in *mta* was much higher compared with WT (**S4 Fig**). In addition, a total of 261 TFs including MYB, bHLH and other proteins, such as MATE, WRKY, NAC, and ethylene-responsive, auxin-responsive, light-inducible proteins, and fatty acyl-CoA reductase (FAR), ribulose bisphosphate carboxylase/oxygenase activase A, were identified as participating in chloroplast biosynthesis and movement, pigment biosynthesis, and stress response (**S3 Table**). The transcriptome analysis was consistent with the phenotypes of *mta* and WT. These results suggest that differential expression of genes might affect chloroplast development and chlorophyll biosynthesis, and thus contributed to the albino leaf phenotype of *mta*.

**Table 4:** DEGs and TFs involved in chloroplast development, and chlorophyll biosynthesis in *mta* transcriptome.

Function	Gene or TF family	Expression level in *mta*	Annotation
Chloroplast development	PIF3	Up-regulated	Transcription factor PIF3
	GLK	Down-regulated	Transcription factor GLK1
Chloroplast division	FtsZ	Down-regulated	Cell division protein, chloroplastic
Chlorophyll biosynthesis	*HEMA*1	Down-regulated	Glutamyl-tRNA reductase 1, chloroplastic
	*HEME*1	Up-regulated	Heme oxygenase 1, chloroplastic
	*CHLD*	Down-regulated	Magnesium-chelatase subunit ChlD, chloroplastic
	GUN4	Down-regulated	Tetrapyrrole-binding protein, chloroplastic
	*CHLI*1	Down-regulated	Magnesium-chelatase subunit ChlI, chloroplastic
	*CRD*1	Down-regulated	Magnesium-protoporphyrin IX monomethyl ester
	*PORA*	Down-regulated	Protochlorophyllide reductase A, chloroplastic
	*PORB*	Down-regulated	Protochlorophyllide reductase B, chloroplastic
Pigment biosynthesis	ARR2	Up-regulated	Two-component response regulator ARR2
	*CHS*	Up-regulated	Chalcone synthase
	*DFR*	Up-regulated	Dihydroflavonol-4-reductase
Transcription factors	MYB	Down-regulated	Myb-related protein
	bHLH	Down-regulated	Transcription factor bHLH
	MATE	Up-regulated	MATE efflux family protein 5
	WRKY	Up-regulated	WRKY transcription factor
	NAC	Up-regulated	NAC transcription factor
	IAA	Down-regulated	Auxin-responsive protein

### Proteomic analysis

#### Chloroplast protein preparation and 2D-DIGE analysis

Protein sample preparation is a critical step in proteomic analysis. Chloroplast organelles were isolated and subsequently observed by a fluorescence microscope. Consistent with the chloroplast ultrastructure observation (Fig 2), chloroplasts in WT had a spindle shape, and the chloroplasts in *mta* were inflated and fewer in number (S5 Fig). The Bradford method was used to quantify analysis whole leaves chloroplast proteins. 2D-DIGE was used to identify DEPs. Coomassie blue G-250 stained proteins were directly matched to CyeDye images of analytical gels (S6 and S7 Figs). Approximately 1,645 spots were selected and 100 differential spots were excised for MS analysis. A total of 48 proteins including 33 down-regulated and 15 up-regulated in *mta*, were identified from chloroplast DEP spots. The size of the chloroplast proteins ranged from 14.4 to 94 kDa, and the isoelectric points (pI) of most of the proteins ranged from 5.08 to 9.4. Proteins with adjusted fold change (average volume ratio) values > 0 were up regulation, otherwise down regulation in *mta* (S4 Table).

#### DEP identification and functional classification

The normalized spot volume for each spot was calculated relative to the internal standard using DeCyder software and reflects protein abundance. Protein identification by MALDI-TOF/TOF MS revealed that a number of DEPs were found in multiple spots. And the 48 DEPs were divided into eleven categories based on functional groups. These categories mainly included photosynthesis, antioxidant/ hydrogen peroxide enzyme, photosynthesis-antenna proteins, component of chloroplast ribosome, chloroplast movement, stress response, oxidation-reduction reaction, photorespiration, protein phosphatase, phospholipid metabolism and other unknown function (Table 5). Most of the DEPs are nuclear-encoded and imported into the chloroplast and thylakoid membrane. These results indicate that the nucleus regulates essential aspects of albino leaf color formation in *mta*.

**Table 5:** Summary of chloroplast proteins and functional annotations.

Functional group	Protein function	Protein name	Coding genome	Subcellular localization
Ⅰ	Photosynthesis/photosystemⅠ/ATP synthase	ATPB, ATPE,ATPA	Chloroplast	T
	Photosynthesis/photosystemⅠ	PsaC	Chloroplast	T
	Photosynthesis/photosystemⅡ	Lhca1, PsbP, PsbO, HCF136, CYP38, PPL2	Nuclear	T
	Photosynthesis/cytochrome b6f complex	PETC, RISP	Nuclear	T
	Photosynthesis/NADPH dehydrogenase complex	NDF4, NDF1, CYP20-2	Nuclear	T
	Photosynthesis/Calvin-Benson cycle	GGT1, RBCS1A, RCA	Nuclear	C
	Photosynthesis/Calvin-Benson cycle	RBCL	Chloroplast	C
Ⅱ	Antioxidant/Hydrogen peroxide enzyme	CAT2	Nuclear	C
	Antioxidant/Dimethylglycine dehydrogenase	GLDP2	Nuclear	C
Ⅲ	Cation channels	VDAC	Nuclear	C
Ⅳ	Embryonic development	EMB140	Nuclear	C
Ⅴ	Component of chloroplast ribosome	RPL12	Nuclear	C
	Chloroplast movement	CHUP1	Nuclear	C
Ⅵ	Stress response	NDPK1	Nuclear	C
Ⅶ	Oxidation-reduction reaction	MOA2.2	Nuclear	C
Ⅷ	Photorespiration	CI76	Nuclear	C
Ⅸ	Protein phosphatase	PAP27	Nuclear	C
Ⅹ	Phospholipid metabolism	ACBP	Nuclear	Unknown
Ⅺ	Unknown	F13M7.10,T7F6.22, T21B4	Nuclear	Unknown

T: Thylakoid membrane; C: Chloroplast.

### Transcriptome and proteome data mining

**Fig 5:**
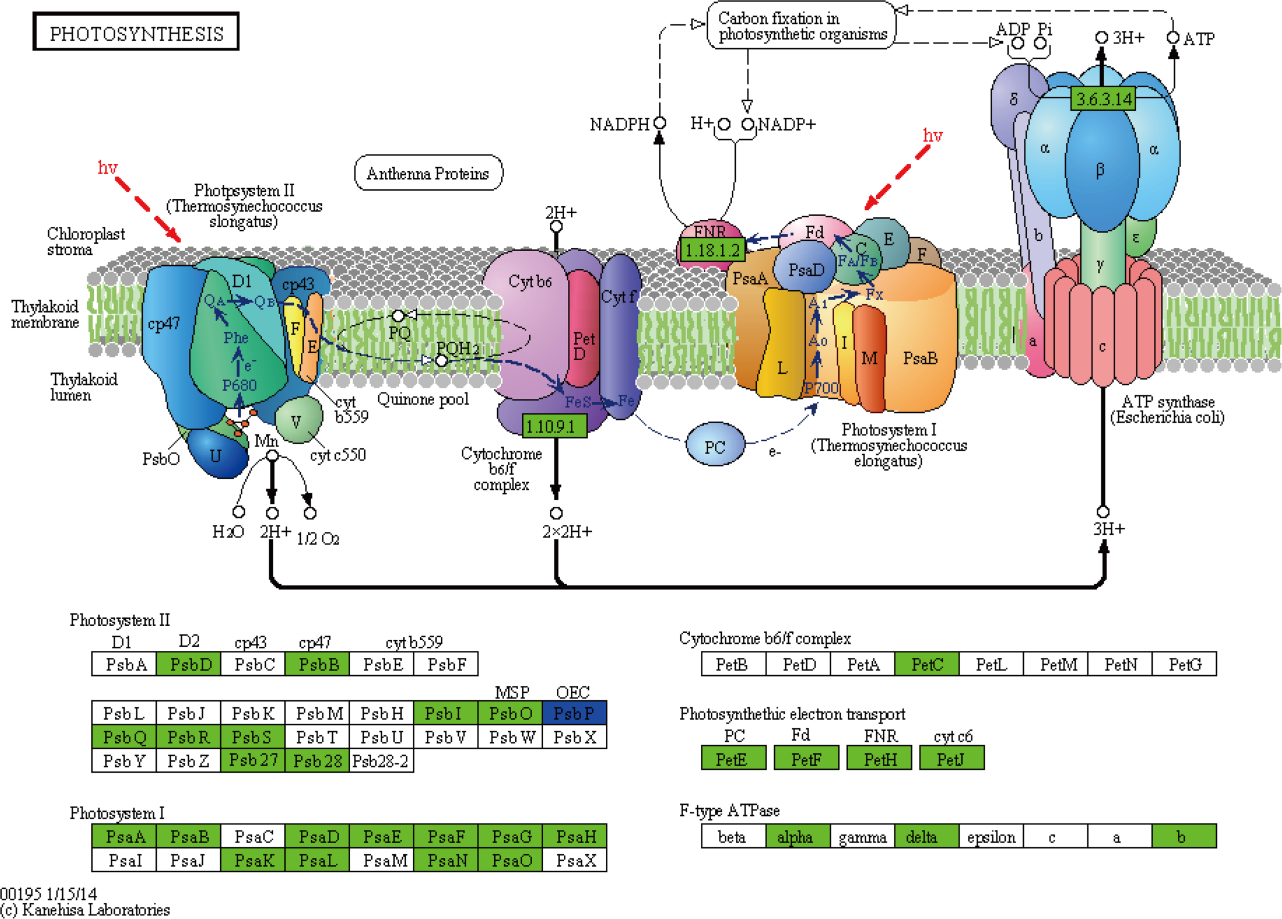
Differentially expressed genes and proteins mapped to photosynthesis pathway. The known pathways were obtained from KEGG database. Green color denotes lower expression in *mta* compared with WT, while red color denotes higher expression. Blue color denotes both up- and down-regulated genes in *mta* compared to WT.

**Fig 6:**
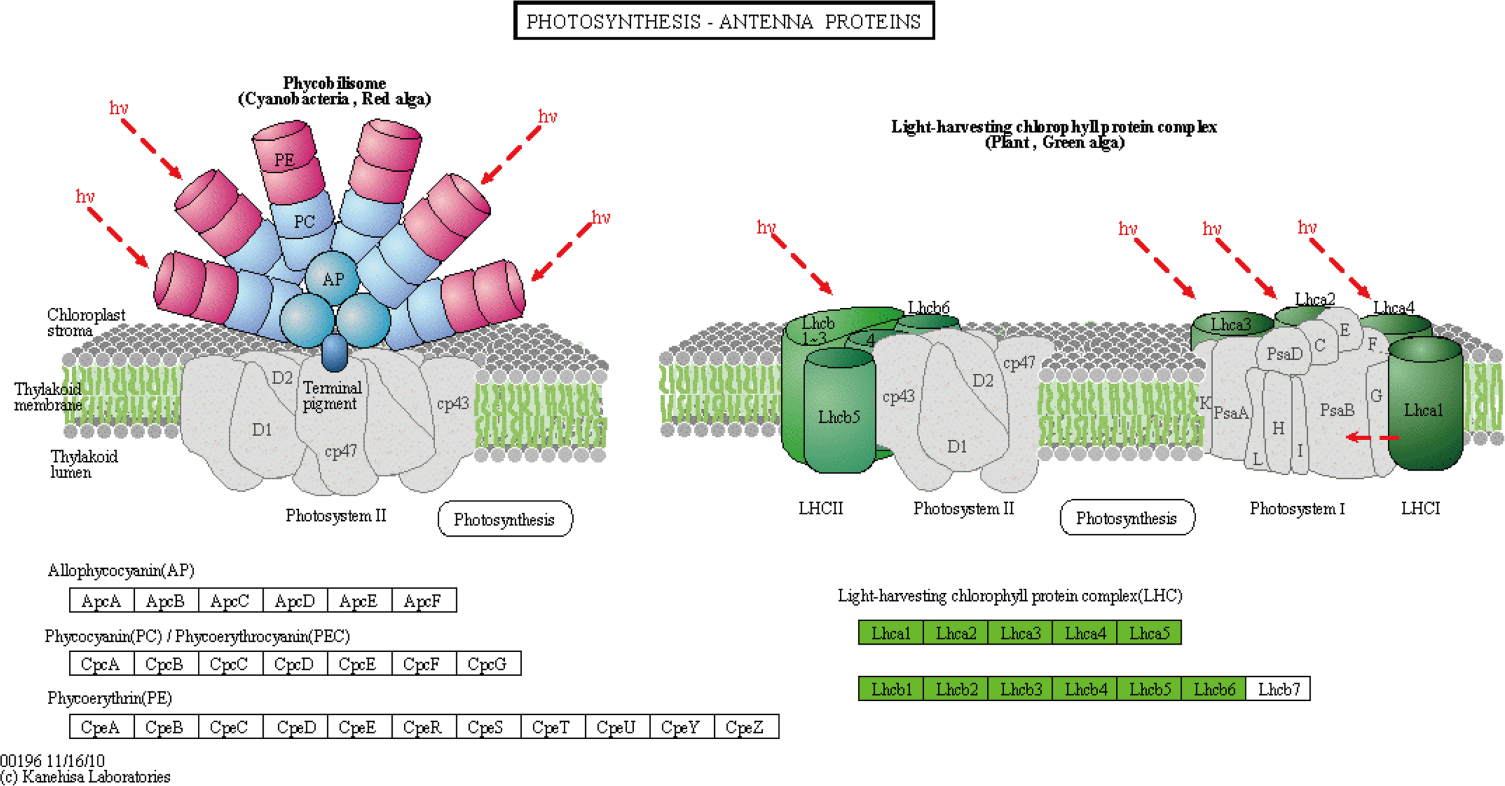
Differentially expressed genes and proteins mapped to photosynthesis-antenna proteins pathway. Green color denotes lower expression in *mta* compared with WT.

Integrative analysis of the transcriptome and the chloroplast proteome data provided an important tool for verifying the expression of key genes in *mta*. The distribution of DEGs and DEPs among species was counted based on NCBInr (**S8 Fig**). The close matches were from *Triticum aestivum*, *Aegilliops tauschii*, *Triticum urartu*, *Hordeum vulgare*, *Oryza sativa*, *Zea mays*, and *Arabidopsis thaliana*. To reveal the correlation between DEGs and DEPs precisely, the genes and proteins coded by them were combined analyzed. The photosynthesis pathway contained 78 DEGs, 24 genes of them were in photosystem I (PSI), 28 genes were in PSII, two genes were in the cytochrome b6/f complex, 18 genes were in the photosynthetic electron transport (**Fig 5**), and six genes were in the light-harvesting chlorophyll protein complex (LHC) (**Fig 6**). The DEPs were also functioned in those items, including one in PSI, six in PSII, two in the cytochrome b6/f complex, three in the F-type ATPase, four in LHC, and three in NADH dehydrogenase complex. The expression levels of all these photosynthetic proteins were lower in *mta* compared to WT. The result was in accordance with the DEGs expression level (**Figs 5 and 6, S4 Table**). The genes and corresponding proteins participated in photosynthesis may be associated with the leaf color variation in *mta*.

Despite those, proteins related to chloroplast, stress response, and other metabolic pathways were identified (**Table 5**). The chloroplast protein-coding genes were located in the nucleus and were functioned as involving in the same pathways. Generally, the relationship between DEGs and chloroplast DEPs expression level was highly consistent with the transcriptome and proteomic analyses. These data further demonstrate that the leaf color formation of spaceflight-induced mutant *mta* is likely to be caused by gene mutation or epigenetic modification, which directly regulate chloroplast development and photosynthesis.

### Verification of selected DEPs and DEGs

**Fig 7:**
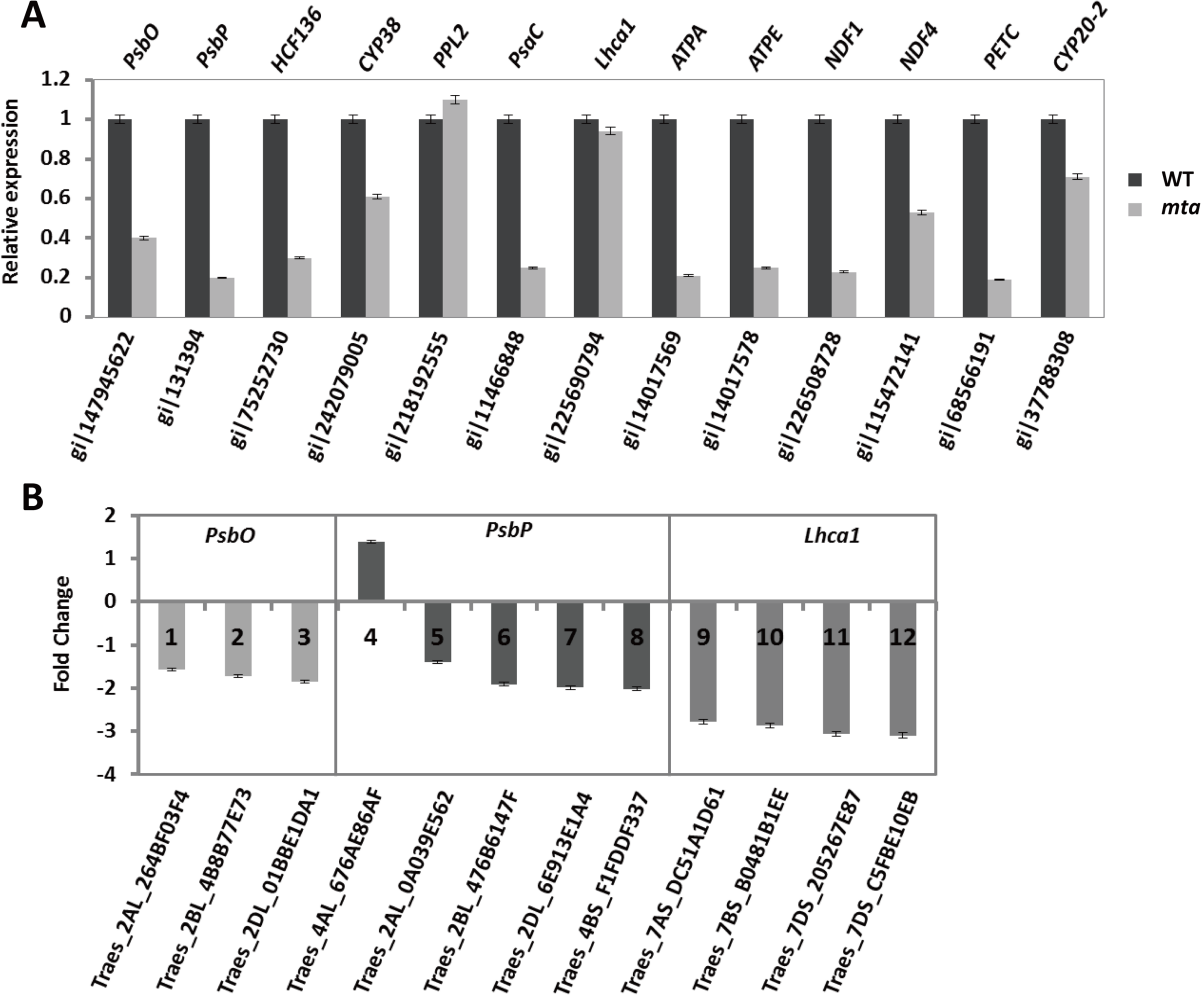
Quantitative real-time PCR analysis of 13 DEP-coding genes. The relative gene expression levels of selected target genes were normalized to a BestKeeper composed of the endogenous reference gene *TaActin*. (A) The black and gray bars represent WT and *mta*, respectively. Low values indicate down-regulation in *mta*, while high values indicate up-regulation. (B) The different colors represent 12 DEGs, including *PsbO* (1–3), *PsbP* (4–8), *Lhca1* (9–12). Positive values indicate higher expression in *mta* and negative values denote higher expression in WT. Error bars represent the standard deviation.

To validate the transcriptome and proteomic analyses, qRT-PCR was performed for a few key photosynthetic genes selected based on DEP expressed sequence tags (EST) and DEGs. Only one proteomic PPL2 expression levels slight varied compared with the transcript levels determined from qRT-PCR analysis. The down-regulation pattern of 12 chloroplast DEPs in *mta*, including PsbO, PsbP, HCF136, CYP38, Lhca, PsaC, ATPA, ATPE, NDF1, NDF4, PETC and CYP20-2 was consistent with the corresponding transcripts based on qRT-PCR (**Fig 7A, S5 Table**). The fold-change values of the 12 photosynthesis-related DEGs, including *PsbO*, *PsbP* and *Lhca1* were largely keeping the same expression levels in *mta* (**Fig 7B**). The verification results demonstrated that the photosynthetic genes showed similar expression patterns between DEPs and DEGs from omics analysis.

## Discussion

During spaceflight, various factors including cosmic irradiation, microgravity and space magnetic fields possibly interact to produce a unique environment that imposes both mutagenic and non-mutagenic stress on plant seeds. Thus, spaceflight has been an effective method for inducing a mutation [42]. The albino-lethal mutant *mta* used in this study and other leaf color mutants were obtained from spaceflight mutagenic induction. We speculated all changes in *mta* were caused by gene mutation or genetic modification. However, the molecular mechanisms leading to leaf color variation exposed to spaceflight in hexaploid wheat chlorophyll deficiency mutants are still not-well known.

The chloroplasts development and photosynthetic pigments are the primary factors affecting leaf color formation in higher plants. To date, leaf color mutants and genes regulating chloroplast development and Chl biosynthesis have been reported in many plant species including *Arabidopsis*, rice, maize and tomato [43–46]. Mutants with dysfunctional chloroplasts usually have leaves that lose their green color. In addition, abnormal plastid number and development may lead to impaired formation of the thylakoid membranes and reduced accumulation of Chl *a*/ *b*- binding proteins of the light-harvesting complexes I and II [46]. Thus, changes in leaf color could reflect the abnormal development and function of the plastid. In this study, the structure and quantity of chloroplasts in WT and *mta* were observed using TEM and fluorescence microscopy, respectively. Our results strongly suggest a relationship between chloroplast morphology and leaf color. The number and shape and inner structure of chloroplasts in *mta* were distinct from those of WT (**Fig 2 and S5 Fig**). Furthermore, the Chl and carotenoid content in *mta* were significantly lower than that in WT. Together, our results show that leaf color variation was directly determined by chloroplast number and development, and Chl biosynthesis. In addition, low temperature stress made a temporary influence on leaf color variation. This might be caused by the effects on the construction of chloroplast thylakoid membranes and changes in carotenoid/Chl content.

Integrating transcriptome and proteome profiling data is generally believed to provide a better understanding of the differences in gene regulation and complex biological processes between wild type and variations [47]. As transcriptome analyses provide information about quantitative changes in gene expression, these data can be used to obtain fundamental insights into specific pathways and genes associated with certain species [13]. In addition, proteomic analysis using approaches such as 2D-DIGE and MALDI-TOF/TOF are helpful in identifying the differences and categories of abundant proteins [26].

Here, we cataloged differences in mRNA and protein abundances that might be associated with leaf color formation in WT and *mta* through integrated profiling of gene expression using RNA-Seq and chloroplast proteomic analyses. We obtained 4,588 DEGs and 48 chloroplast DEPs. Many DEGs annotations were related to chloroplast, chloroplast envelope, thylakoid membrane and stroma. These genes were enriched in KEGG pathways, such as “photosynthesis”, “photosynthesis-antenna proteins” and “porphyrin and chlorophyll metabolism” (**Table 3**). Meanwhile, most of chloroplast DEPs was involved in photosynthesis, followed by component of chloroplast activity and other metabolic pathways (**Table 5**). As in photosynthetic pathway, the DEGs and DEPs were significantly involved in PSI, PSII, cytochrome b6/f complex, F-type ATPase, NADH dehydrogenase complex and LHC. The DEGs and corresponding DEPs showed the same expression level (**Figs 5 and 6**). These results were consistent with our original prediction that leaf color formation is greatly affected by chloroplast development, and photosynthesis. Importantly, this is the first study to provide transcriptome information for a leaf color mutant in hexaploid wheat. Our *mta* chloroplast proteome results are consistent with those of a previously reported wheat chloroplastic proteome [27]. Thus, the unigene from omics data will aid researchers in identifying the specific genes relevant to leaf color formation in *Triticum aestivum* L.

**Fig 8:**
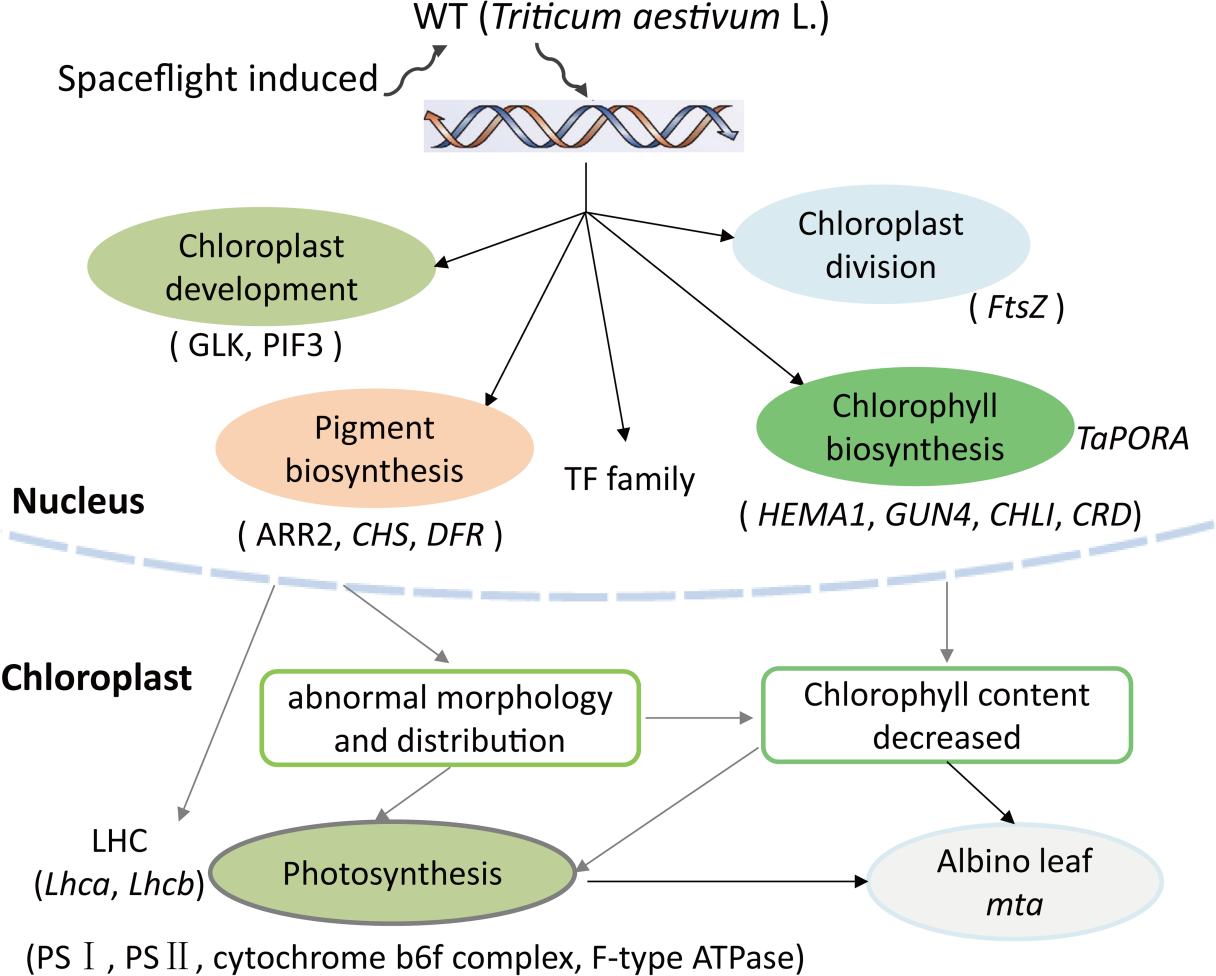
Possible leaf color formation pathways of albino mutant *mta* in wheat (*Triticum aestivum* L.).

Previous studies have shown that chloroplast development is a complex and highly regulated process that includes light signaling during photo-morphogenesis, the transition from proplastids to chloroplasts, import of nuclear-encoded chloroplast proteins, thylakoid biosynthesis, chloroplast division, and retrograde signaling from the chloroplast to the nucleus [7, 8, 48]. PIF3 and GLK had been identified as important TFs regulating chloroplast development. PIF3 is a repressor that negatively regulates chloroplast development in *Arabidopsis* [18]. PIF3 mediates the initial phases of light-induced chloroplast development through regulation of a subset of rapidly photoresponsive nuclear genes encoding plastid and photosynthesis-related components [19]. GLK is required for the expression of nuclear photosynthetic genes and chloroplast development in diverse chlorophyll deficiency plant species, including maize, rice, and *Arabidopsis* [2, 20–22]. FtsZ is a key structural component of the chloroplast division machinery in perhaps all photosynthetic eukaryotes [23]. The mechanism of Chl biosynthesis and most the genes involved have been identified in *Arabidopsis* [10]. In rice, *OsPORB* is essential for maintaining light-dependent Chl synthesis throughout leaf development, whereas *OsPORA* mainly functions in the early stages of leaf development [17]. Despite those, many other transcription factors may be potentially regulated the leaf color formation or plant growth and development. For example, auxin-responsive factor acts indirectly to regulate chloroplast movements [49]. MYB family is responsible for the biosynthesis of phenylpropanoids, including anthocyanin and phlobaphene pigments [50]. Consistent with what is known about these genes and TF family, in our study, we found several genes and TFs regulating chloroplast development and division, Chl biosynthesis, and pigment biosynthesis (**Table 4 and S2 Table**). The TF family contained GLK, repressor PIF3, FtsZ, ARR2 and other transcription factors, such as MYB, bHLH, WRKY, NAC, and auxin-responsive factor. The related genes included *HEMA1*, *GUN4*, *PORA*, *PORB*, *CHS*, and *DFR*. The expression level and functional annotations of all these genes provide support for our hypothesis that leaf color variation is determined by combined regulation of multiple genes. We also suppose that *TaGLK*, *TaPIF3*, and *TaFtsZ* may regulate chloroplast development and division, while *TaHEMA*, *TaCHLD*, *TaPORB*, and *TaPORA* participate in chlorophyll biosynthesis in wheat. Changes in these genes expression may affect photosynthesis and all together lead to the albino phenotype in *mta* (**Fig 8**).

To find the cause of decreased chlorophyll content in *mta*, we chose one gene *TaPORA* and detected the promoter methylation level. We found *TaPORA* promoter methylation level was much higher in *mta* compared to WT (**S4 Fig**). Previous studies have shown that plant genomes are characterized by a relatively high degree of nuclear DNA methylation [51], and methylation in promoters is responsible for low gene expression or silencing [52, 53]. In addition, other research indicates that spaceflight induces both transient and heritable alterations in DNA methylation and gene expression in rice [54]. We speculate that spaceflight environment might induce heritable alteration in *TaPORA* promoter methylation in *mta*. And the promoter methylation might play important role in the *TaPORA* lower expression. However, we found many SNPs existed between WT and *mta*. These SNPs uniformly distributed on the 21 wheat chromosomes within gene or Intergenic regions (**S9 Fig**). We also surmise that gene mutation in the nucleus is the main factor controlling the expression of albino trait in *mta*, which should be investigated in the future study.

## Conclusions

In conclusion, the chloroplast structure and photosynthetic pigments content in the spaceflight-induced mutant *mta* were significantly different from those in WT H6172 (*Triticum aestivum* L.). Transcriptome and chloroplast proteomic analyses of WT and *mta* revealed that DEGs and DEPs were mainly involved in chloroplast activities, and photosynthesis pathways. In addition, these DEGs and corresponding DEPs showed similar expression pattern. In addition, genes participated in chloroplast development and division, chlorophyll and pigment biosynthesis were identified from albino mutant *mta* and WT transcriptome database. qRT-PCR verified that those photosynthetic DEGs and DEPs were differentially expressed in *mta*. Based on these results, we speculate that changes in chloroplast development, Chl biosynthesis, and the protein levels of photosynthetic proteins all contribute to the differences in leaf color formation in the *mta* (**Fig 8**). We also show that spaceflight might be used of mutagenesis in crop breeding.

## Supporting information

S1 Fig The outline of 2D-DIGE from proteomic analysis. (PDF)

S2 Fig The ratios of quantified transcripts were plotted between two biological replicates. (A) wild type samples (WT_R1 and WT_R2) and (B) albino mutant samples (*mta*_R1 and *mta*_R2).
(PDF)

S3 Fig DEGs mapped to the porphyrin and chlorophyll metabolism pathway. (PDF)

S4 Fig Bisulfite sequencing PCR (BSP) validation of *TaPORA* promoter methylation. (A) CpG island distribution in the *TaPORA* promoter methylation, BSP primer design regions and sequencing data. (B) *TaPORA* promoter methylation level. (C) *TaPORA* expression determined level by qRT-PCR analysis.
(PDF)

S5 Fig Fluorescence microscope visualization of chloroplasts. (a, c) Chloroplasts visualized under white light, (b, d) chloroplast visualized under a fluorescence from High Pressure Mercury (HPM) lamp. (1) large quantity of chloroplasts; (2) broken chloroplasts; (3) the size of intact chloroplasts in *mta* is bigger than in WT. Magnification bar = 20 μm.
(PDF)

S6 Fig Expression profiles of proteins and three-dimensional expression maps of chloroplast protein spots. (PDF)

S7 Fig 2D-DIGE gel images of leaf total proteins. (a, b, c) Three replicated experiments of the internal standard; (d, e, f) three replicated experiments of WT chloroplast proteins; (g, h, i) three replicated experiments of *mta* chloroplast proteins. The blue, red and green gel images were labeled with fluorescent Cy2, Cy5 and Cy3 dyes, respectively.
(PDF)

S8 Fig DEGs and chloroplast DEPs homologous species distribution based on BLAST searches against NCBInr. (PDF)

S9 Fig The number of SNPs and genes with SNP in 21 wheat chromosomes. Blue color represents number of SNPs, and red color represents number of genes with SNP.
(PDF)

S1 Table Summary of transcriptome WT and mta sequencing data. Q30 (base content of clean reads with a quality no less than 30).
(XLSX)

S2 Table The expression and annotation of DEGs associated with leaf color formation. (XLSX)

S3 Table Summary of differentially expressed transcription factors in *mta* / WT. (XLSX)

S4 Table Features of the 48 chloroplast proteins identified by MALDI-TOF MS from *mta* / WT. ^a^ Spot no are given in **S6 Fig**. ^b^ Fold change: average volume ratio (mta/ WT). ^c^ Mascot score: more than 20 is significant (P≤0.05) from MS data. ^d^ SC: sequence coverage. ^e^ Molecular weights (MW, kDa) and isoelectric point (pI) of spots from the gel.
(XLSX)

S5 Table Primers for bisulfite sequencing PCR (BSP) analysis. (XLSX)

S6 Table Primers for qRT-PCR analysis. (XLSX)
