# Supplementary figures and images for: Transcriptome and proteomic analyses reveal multiple differences associated with chloroplast development in the spaceflight-induced wheat albino mutant mta
Source: PLoS One. 2017 May 24;12(5):e0177992. doi: 10.1371/journal.pone.0177992 (PMC5443577; doi:10.1371/journal.pone.0177992)

S1 Fig

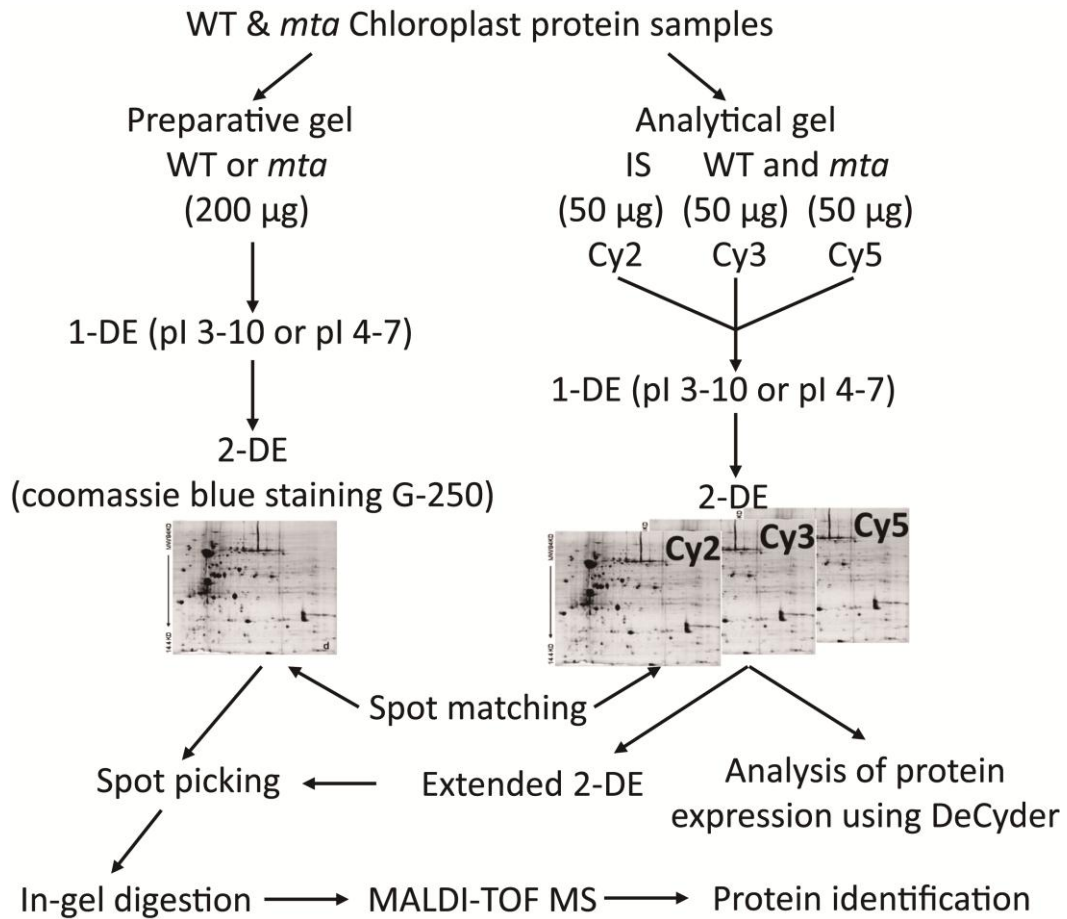

Supplement: S1 Fig — (PDF) [file pone.0177992.s001.pdf]

S2 Fig

**A**

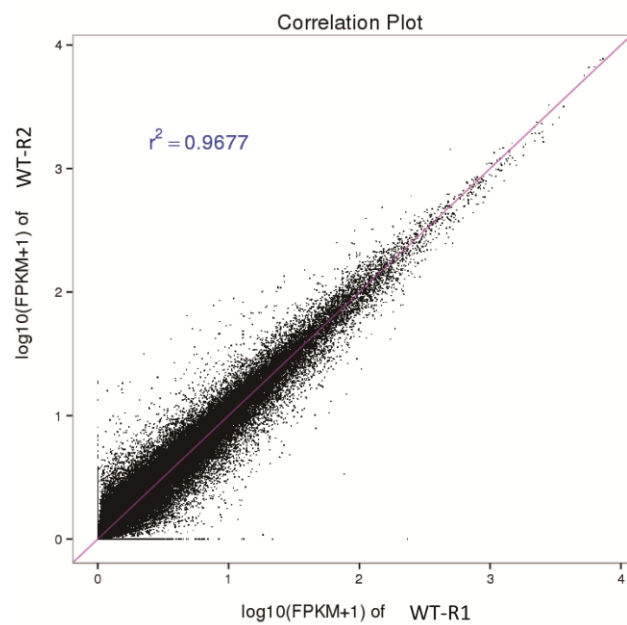

**B**

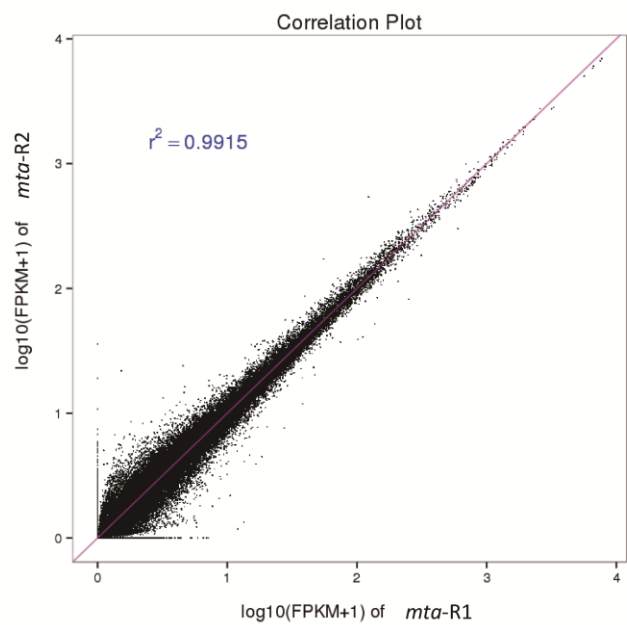

Supplement: S2 Fig — (A) wild type samples (WT_R1 and WT_R2) and (B) albino mutant samples (mta_R1 and mta_R2). (PDF) [file pone.0177992.s002.pdf]

**S3 Fig**

## PORPHYRIN AND CHLOROPHYLL METABOLISM

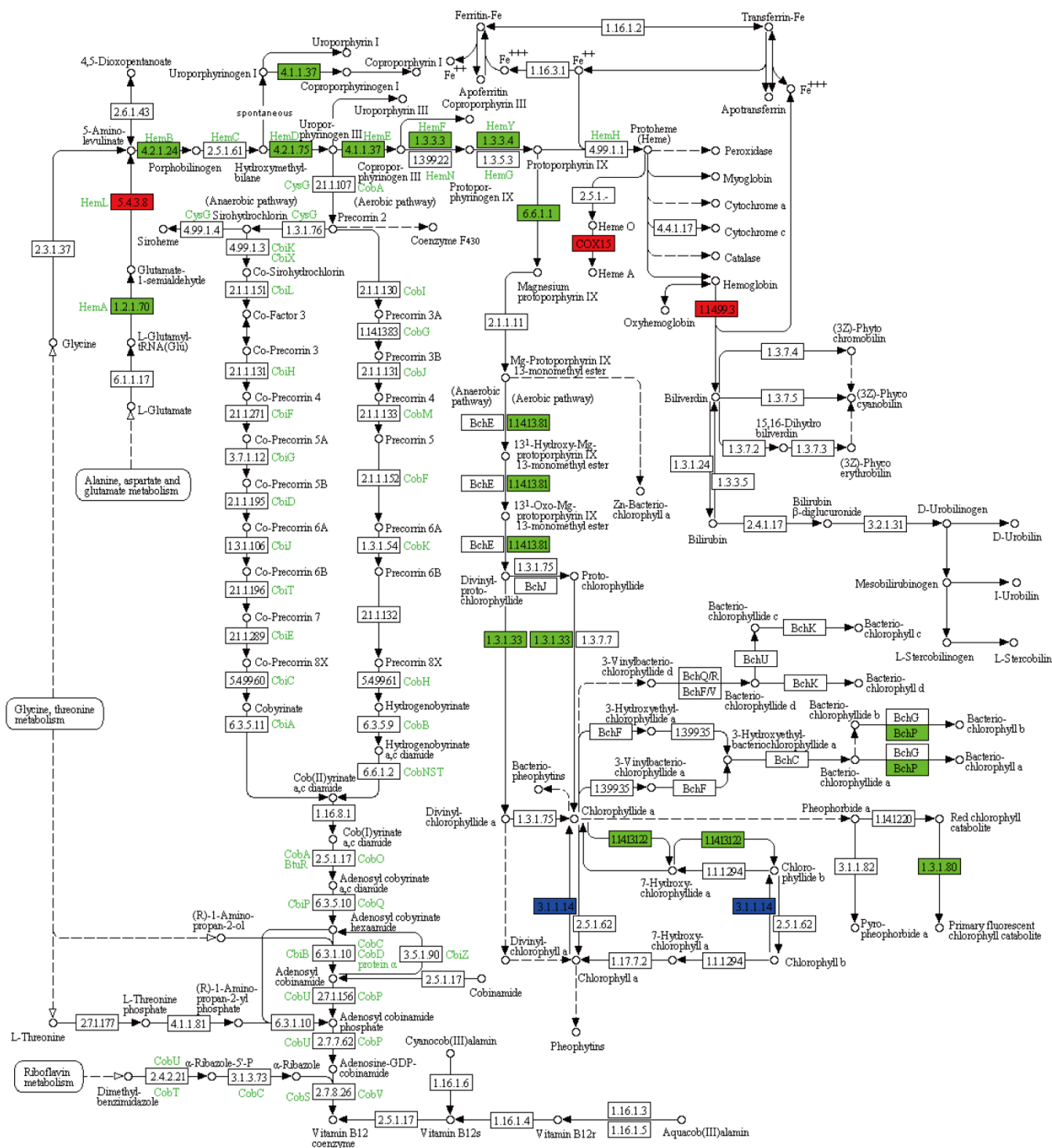

Supplement: S3 Fig — (PDF) [file pone.0177992.s003.pdf]

S4 Fig

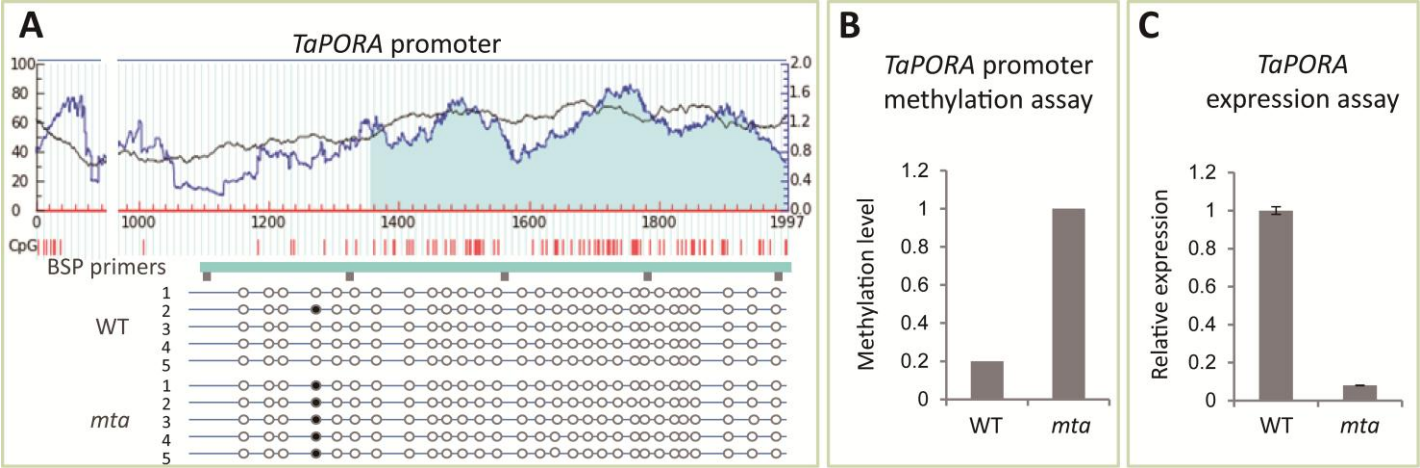

Supplement: S4 Fig — (A) CpG island distribution in the TaPORA promoter methylation, BSP primer design regions and sequencing data. (B) TaPORA promoter methylation level. (C) TaPORA expression determined level by qRT-PCR analysis. (PDF) [file pone.0177992.s004.pdf]

S5 Fig

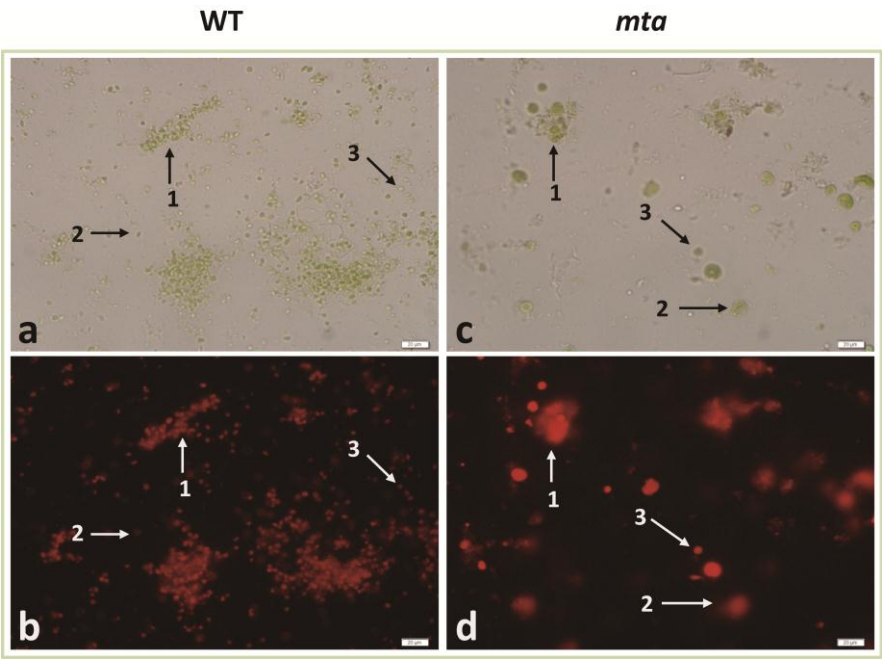

Supplement: S5 Fig — (a, c) Chloroplasts visualized under white light, (b, d) chloroplast visualized under a fluorescence from High Pressure Mercury (HPM) lamp. (1) large quantity of chloroplasts; (2) broken chloroplasts; (3) the size of intact chloroplasts in mta is bigger than in WT. Magnification bar = 20 μm. (PDF) [file pone.0177992.s005.pdf]

S6 Fig

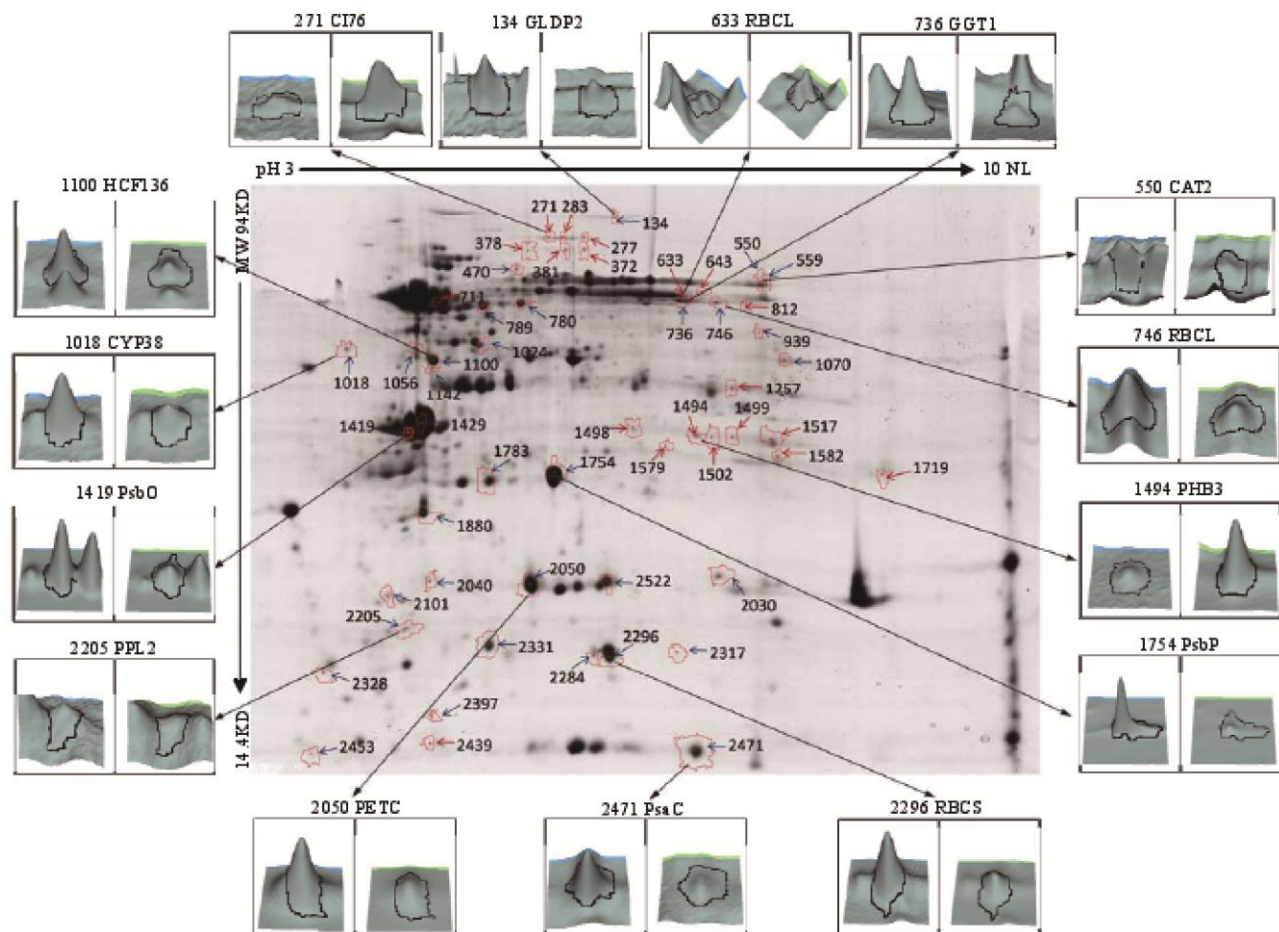

Supplement: S6 Fig — (PDF) [file pone.0177992.s006.pdf]

S7 Fig

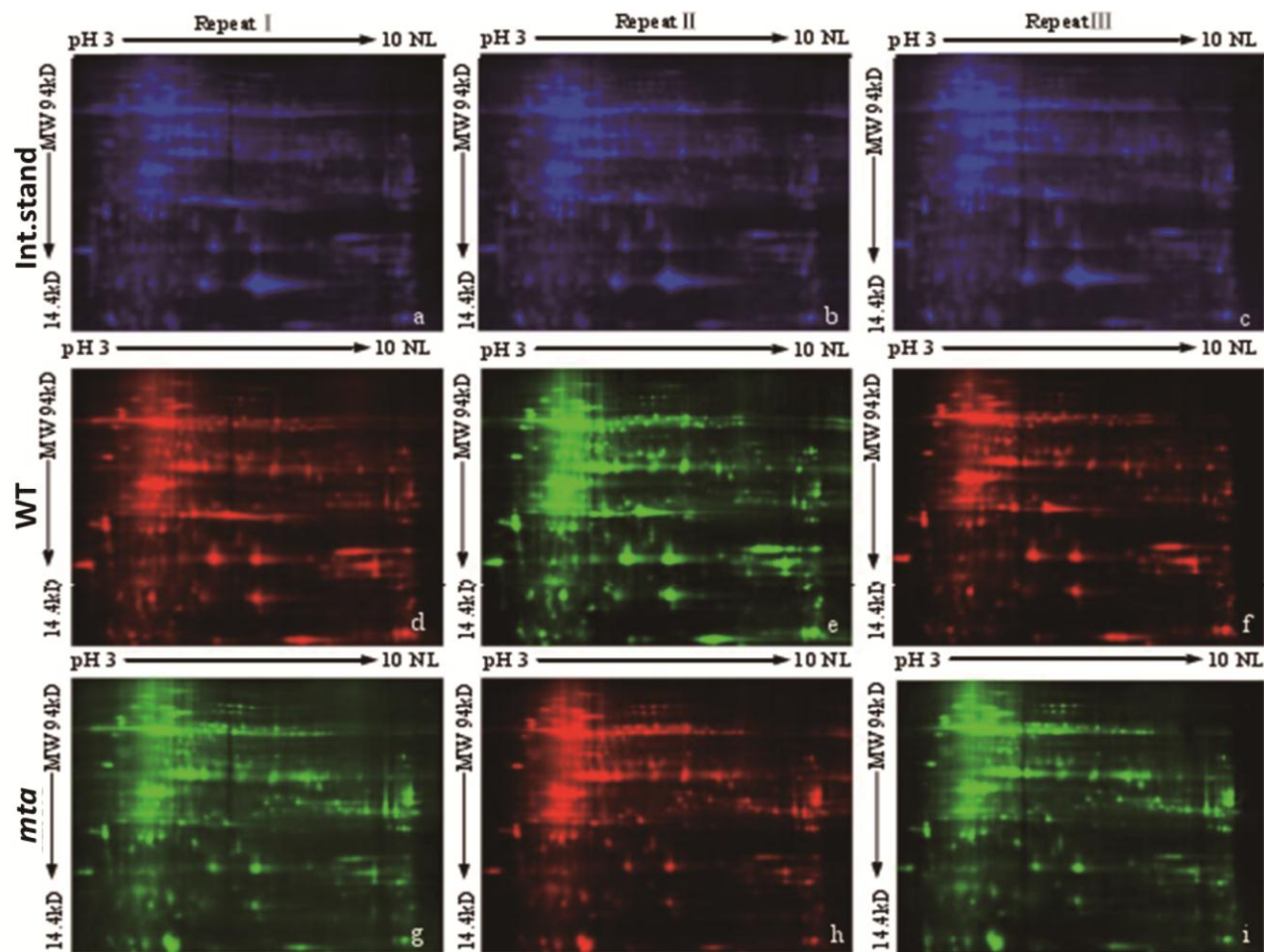

Supplement: S7 Fig — (a, b, c) Three replicated experiments of the internal standard; (d, e, f) three replicated experiments of WT chloroplast proteins; (g, h, i) three replicated experiments of mta chloroplast proteins. The blue, red and green gel images were labeled with fluorescent Cy2, Cy5 and Cy3 dyes, respectively. (PDF) [file pone.0177992.s007.pdf]

S8 Fig

A

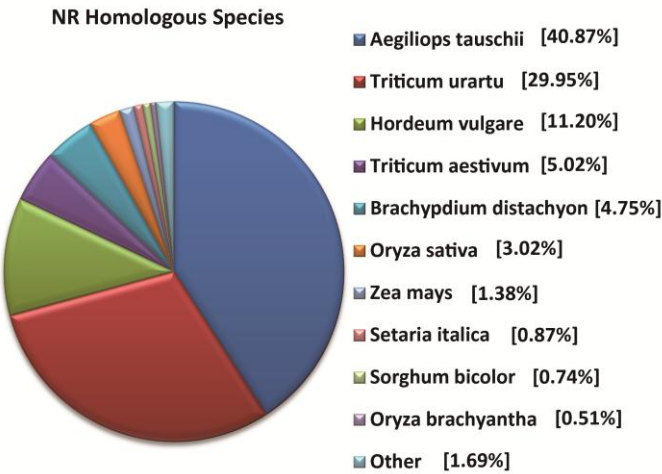

B

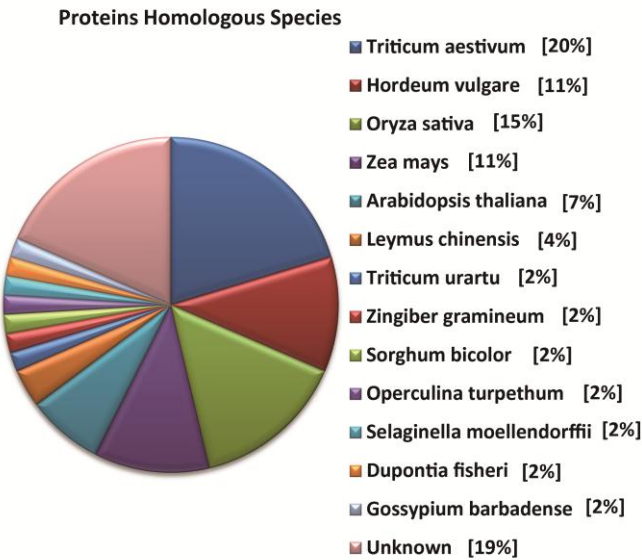

Supplement: S8 Fig — (PDF) [file pone.0177992.s008.pdf]

S9 Fig

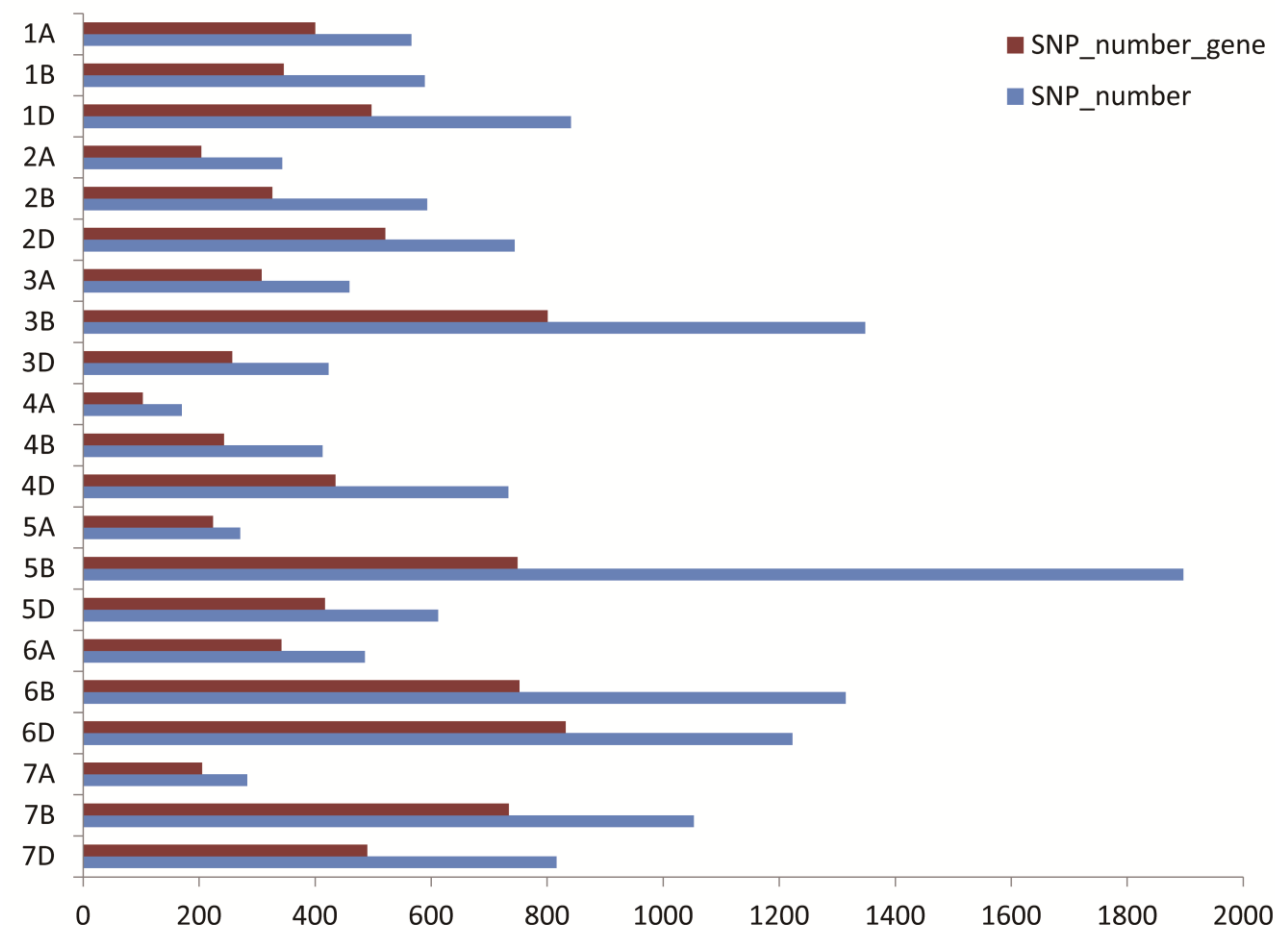

Supplement: S9 Fig — Blue color represents number of SNPs, and red color represents number of genes with SNP. (PDF) [file pone.0177992.s009.pdf]
